# Exploring the content and delivery of feedback facilitation co-interventions: a systematic review

**DOI:** 10.1186/s13012-024-01365-9

**Published:** 2024-05-28

**Authors:** Michael Sykes, Zahava R. S. Rosenberg-Yunger, Matthew Quigley, Lavanya Gupta, Owen Thomas, Lisa Robinson, Karen Caulfield, Noah Ivers, Sarah Alderson

**Affiliations:** 1 Northumbria University, Newcastle Upon Tyne, UK; 2 Toronto Metropolitan University, Toronto, Canada; 3 Monash University, Melbourne, Australia; 4 University of Leeds, Leeds, UK; 5 Newcastle Upon Tyne NHS Foundation Trust, Newcastle Upon Tyne, UK; 6 University of Toronto, Toronto, Canada

## Abstract

**Background:**

Policymakers and researchers recommend supporting the capabilities of feedback recipients to increase the quality of care. There are different ways to support capabilities. We aimed to describe the content and delivery of feedback facilitation interventions delivered alongside audit and feedback within randomised controlled trials.

**Methods:**

We included papers describing feedback facilitation identified by the latest Cochrane review of audit and feedback. The piloted extraction proforma was based upon a framework to describe intervention content, with additional prompts relating to the identification of influences, selection of improvement actions and consideration of priorities and implications. We describe the content and delivery graphically, statistically and narratively.

**Results:**

We reviewed 146 papers describing 104 feedback facilitation interventions. Across included studies, feedback facilitation contained 26 different implementation strategies. There was a median of three implementation strategies per intervention and evidence that the number of strategies per intervention is increasing. Theory was used in 35 trials, although the precise role of theory was poorly described. Ten studies provided a logic model and six of these described their mechanisms of action. Both the exploration of influences and the selection of improvement actions were described in 46 of the feedback facilitation interventions; we describe who undertook this tailoring work. Exploring dose, there was large variation in duration (15–1800 min), frequency (1 to 42 times) and number of recipients per site (1 to 135). There were important gaps in reporting, but some evidence that reporting is improving over time.

**Conclusions:**

Heterogeneity in the design of feedback facilitation needs to be considered when assessing the intervention’s effectiveness. We describe explicit feedback facilitation choices for future intervention developers based upon choices made to date. We found the Expert Recommendations for Implementing Change to be valuable when describing intervention components, with the potential for some minor clarifications in terms and for greater specificity by intervention providers. Reporting demonstrated extensive gaps which hinder both replication and learning. Feedback facilitation providers are recommended to close reporting gaps that hinder replication. Future work should seek to address the ‘opportunity’ for improvement activity, defined as factors that lie outside the individual that make care or improvement behaviour possible.

**Review registration:**

The study protocol was published at: https://www.protocols.io/private/4DA5DE33B68E11ED9EF70A58A9FEAC02.

**Supplementary Information:**

The online version contains supplementary material available at 10.1186/s13012-024-01365-9.

Contribution to the literature

- Feedback facilitation delivered alongside audit and feedback is recommended to increase improvement in care.
- We describe the content and delivery of feedback facilitation within randomized controlled trials and identify design choices.
- Feedback facilitation is a heterogenous intervention that uses different implementation strategies. The number of strategies being used is increasing. The target of these strategies varies from direct impact upon care to seeking to implement improvement activities. We describe the use of theory logic models and the enactment of tailoring.
- The ability to replicate a study underpins implementation science and impact. Few studies provide sufficient detail to enable replication.

## Background

Audit and feedback is a complex intervention that involves the delivery of feedback on performance over a specific period [1]. Health professionals may not have the knowledge and skills to engage and respond to feedback, and this may create variation in the effectiveness of audit and feedback [2, 3]. Health systems are investing in quality improvement support to feedback recipients [4, 5]

Brown and colleagues [2] describe quality improvement co-interventions as supporting feedback recipients, “to identify the reasons for and develop solutions to sub-optimal performance” (p16). Quality improvement support is a form of feedback facilitation that might help recipients to identify, “barriers and enablers for making change” [6; p3]. The identification of influences and selection of actions to address these is known as ‘tailoring’ [7]. In addition to tailoring, authors have described the need to develop commitment, as the shared resolve to implement a change [8]; for example, through describing implications of current audit performance [9].

There is a lack a clarity about the content and delivery of feedback facilitation. Facilitation is associated with enabling and making a target behaviour easier. In the context of audit and feedback, facilitation might include how to use feedback, undertake quality improvement or set goals and plans [10]. Beyond feedback-specific facilitation, Richie and colleagues describe 22 implementation facilitation skills, including engaging stakeholders, problem-identification/solving and education skills. The template for intervention description and replication (TIDieR) [11] provides a guide to describe the content of interventions. TIDieR highlights the importance of describing what is delivered and why, who delivers the intervention, how, where, when and how much, whether there is tailoring, modifications and if fidelity is assessed and delivered. In relation to what is delivered, the Expert Recommendations for Implementing Change (ERIC) [12] describes 73 different types of implementation intervention. In relation to why a particular intervention is delivered, Colquhoun and colleagues [13] described gaps in the use of theory within audit and feedback interventions. Feedback facilitation could be considered an implementation strategy. Within the current manuscript we will refer to feedback facilitation as an intervention (Table 1), to be consistent with the description of multi-faceted interventions [1, 2], co-interventions [2] and complex interventions [14], and to reflect that feedback facilitation may be composed of multiple implementation strategies.

**Table 1:** Definitions for key terms

Capability – the individual’s psychological and physical capacity to engage in the activity concerned, for example, having the necessary knowledge and skills to perform a particular behaviour [15]
Feedback facilitation – Interventions that seek to make the response to feedback easier. It might contain one or more strategies, for example, training about how to use feedback, set goals or make plans
Feedback recipients – People who receive audit results, for example, health care workers
Implementation strategy—Methods or techniques used to enhance the adoption, implementation, and sustainability of a clinical program or practice [12]
Implication of performance – Consequences from the current quality of care, as described within the audit feedback, for example, implications for patient outcome or service cost
Influence upon performance – Often referred to as determinants, but allowing for a less linear relationship with performance, for example, staff knowledge, beliefs about capability, resources. May serve as barrier or facilitator to performance
Intervention—An action or programme that aims to bring about identifiable outcomes [16]
Opportunity – “all the factors that lie outside the individual that make the behaviour possible or prompt it”, incorporating: “physical opportunity afforded by the environment and social opportunity afforded by the cultural milieu that dictates the way that we think about things” [15]. Examples might include protected time, clinic space, team consensus
Mechanism—A causal link between an exposure (e.g., to some feature of an intervention) and an outcome [14]
Moderator—Factor that increases or decreases the level of influence of an intervention or strategy, for example, connectivity within the organisational network structure [17]
Motivation – “all those brain processes that energize and direct behaviour, not just goals and conscious decision-making. It includes habitual processes, emotional responding, as well as analytical decision-making.” [15]
Pre-disposing condition—Factor that is necessary in order for an implementation mechanism to be activated [18], for example, self-efficacy
Tailoring—The identification of influences and selection of actions to address these influences [7]

Multiple authors (e.g. [18–20]) recommend using logic models to describe the programme theory for an intervention. Describing the intervention components enables replication across contexts with fidelity of identified core components [14]. Lewis and colleagues [17] describe potential components in the causal pathway of interventions: intervention mechanism(s); context; pre-conditions and/or moderators; proximal and distal outcomes. Such frameworks provide a further lens through which to describe the content and delivery of feedback facilitation interventions.

The effectiveness of audit and feedback with or without a feedback facilitation co-intervention is being explored during the updated Cochrane review of randomised controlled trials. Understanding the content and delivery, as well as the effectiveness, of an intervention is extremely valuable and supports the interpretation and use of the findings. The aim of the current study is to describe the content and delivery of feedback facilitation co-interventions used in trials of audit and feedback.

## Method

We explored the content of feedback facilitation co-interventions reported in randomised controlled trials of audit and feedback (A&F). Feedback facilitation trials were identified from the latest update of the Cochrane review of audit and feedback. Within the Cochrane review, co-interventions were described as a form of feedback facilitation, which “could be training about how to use feedback, or to do quality improvement in the practice, or set goals and plans, etc.” [10].

The search criteria and identification of studies is reported by the Cochrane review [21] team, who provided the papers identified as containing feedback facilitation. We reviewed these papers and their citations for further details describing the intervention content.

Inclusion criteria: Papers describing interventions delivered in randomised controlled trials of audit and feedback with additional feedback facilitation co-intervention delivered to health care workers. There were no exclusion criteria.

### Participants

Audit and feedback and/or feedback facilitation developers and/or deliverers.

### Intervention

Feedback facilitation co-interventions delivered alongside audit and feedback.

### Quality assessment

Quality was assessed as part of the Cochrane review.

### Data collection and management

We extracted data from papers describing the trial, from publicly available protocols and from companion papers. Eight reviewers extracted data from the included studies using a specifically designed and piloted proforma adapted from the TIDieR framework [11]. The adapted proforma extended TIDieR to capture the form of implementation strategy [12], theory and logic model, the identification of influences upon performance and work to align improvement actions to influences, whether information to describe the implications of performance was reported and the level of change sought.

The adapted proforma also enabled us to explore whether and how the feedback facilitation co-interventions supported teams to tailor their response to feedback. In identifying whether the facilitation explored influences upon performance, we looked for whether influences or causes were given, sought by data recipients or not recorded. The extraction guide (Appendix A) provided the example of using a framework to identify determinants or other potential list of influences from which recipients selected. The data extractors described the procedure to explore influences using language similar to that in the text and categorised this as ‘sought by data recipients’, ‘given by study team’, ‘co-produced’ or ‘not recorded’. The extractors then described the presence or absence of a process by which implementation strategies were determined; for example, whether they were given by the study team or selected by data recipients.

#### Data was recorded and managed in Excel

Duration of feedback facilitation was calculated in minutes; where specified in days, duration was converted to 450 min per day. Maximum duration was used unless an average time was given. The deliverer of facilitation was classified into expert, peers or improvement specialists [22].

We developed and piloted reviewer guidance notes to accompany the proforma (Appendix A). Each paper was reviewed by 2 reviewers. The reviewers were health service researchers, five of whom were also clinicians. Six reviewers were involved in the development of the codebook through iterative discussion, design and testing. Two further reviewers received training and supervision in use of the code book. The reviewers extracted separately, and disagreements were resolved through consensus between the two reviewers.

### Data analysis and synthesis

Two members of the team (MS and SA, both experienced implementation scientists) cleaned the data set and used the extracted data to codify the ERIC strategies, referring to source papers where necessary. MS and SA analysed the data narratively, graphically and statistically using Excel and StataMP 17. Our analysis drew upon the full data set, with the exception of the narrative analysis of the use of theory, which focussed on the period since a review of the use of theory in audit and feedback [13]. Statistical analysis involved a linear regression to determine if the number of TIDieR framework items not reported changed with publication year. We examined plots of residuals from the regression analyses and performed a Breusch-Pagan test for heteroskedasticity. The synthesis was presented to the research team for challenge. We summarised the content of feedback facilitation interventions and drew upon guidance and wider literature to consider implications for research and practice. To support feedback facilitation providers, we made the different forms of content and delivery that we identified explicit as a series of design choices.

The protocol for this review has been published separately [23]. We report upon variations from the protocol in the discussion.

## Results

**Fig. 1:**
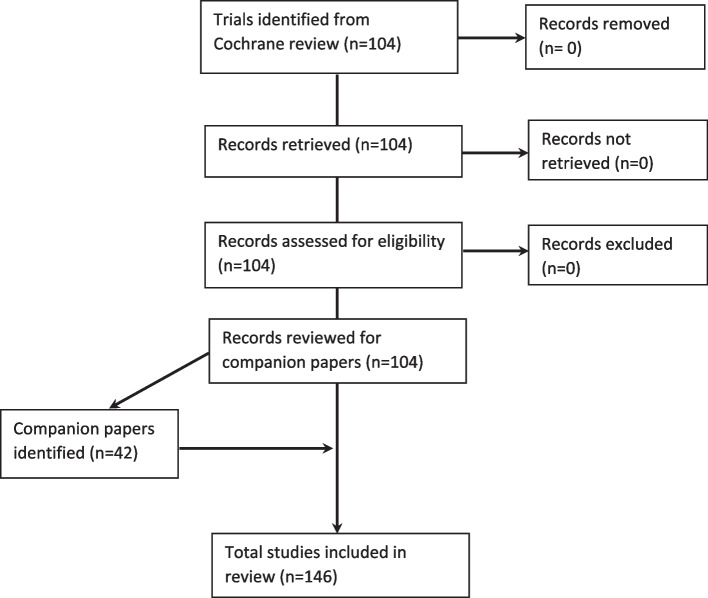
The PRISMA flowchart

The Cochrane review identified 104 randomised controlled trials that delivered feedback facilitation alongside audit and feedback. We included 146 papers describing these trials, as detailed in the below flowchart (Fig. 1).

Table 2 summarises the content and delivery of feedback facilitation in 104 trials. Additional data is provided in the Supplementary Materials. Table 3 presents a cumulative summary of the content across included studies.

**Table 2:** A summary of the content and delivery of the included feedback facilitation interventions

**Trial paper first author + year** [Associated papers]	**Setting**	**Expert Recommendations for Implementing Change (ERIC) strategies** [11]	**Theory used** (Reference, where given)	**Are influences explored?**	**How are actions determined?**	**How is intervention delivered**	**Freq**	**Duration (min)**	**Number of recipients per site**
Althabe, 2019 [24, 25]	Antenatal	Educational outreach + Inform local opinion leaders + Facilitation + Remind clinicians + Change equipment	Diffusion of innovation (Rogers, 1983.); The transtheoretical model of health behaviour change (Prochaska et al., 1997)	Influences given	Given by study team	Face-to-face	18	900	1–2
Avery, 2010 [26]	Primary Care	Educational materials + Educational outreach + Facilitation	Diffusion of innovation (Rogers, 1983; Greenhalgh et al., 2014) Human error theory (Reason, 2000)	Influence sought by data recipients	Not reported	Face-to-face	Not reported	Not reported	Not reported
Awad, 2006 [27]	Primary Care	Educational meeting + Tailor strategies	Not reported	Influence sought by data recipients	Given by study team	Face-to-face + Educational materials	2	Not reported	1–3
Ayieko, 2011 [28, 29]	Secondary care	Educational outreach + Facilitation + Educational materials + Clinical supervision + Change record system	Not reported	Not reported	Given by study team	Face-to-face + Educational materials	7	Not reported	Not reported
Ayieko, 2019 [30, 31]	Secondary care	Implementation blueprint + Educational meeting + Form a learning collaborative + Educational materials + Tailor strategy	Feedback intervention theory (Kluger et al., 1996; Hysong et al., 2012)	Not reported	Not reported	Face-to-face	2	Not reported	6
Baker, 1997 [32]	Primary Care	Remind clinicians	Not reported	Not reported	Determined by recipients	Face-to-face + Educational materials	Not reported	Not reported	1
Baldwin, 2010 [33]	Nursing homes	Educational meeting + Educational materials + Facilitation	Not reported	Not reported	Given by study team	Face-to-face + Educational materials	3	120	Not reported
Barkun, 2013 [34]	Secondary care	Educational materials + Remind clinicians + Educational meeting + Form a learning collaborative + Facilitation + Tailor strategy	Not reported	Co-produced	Given by study team	Face-to-face	3	165	Not reported
Bertoni, 2009 [35]	Primary Care	Educational outreach + Educational materials + Change clinical record system + Change equipment	Not reported	Not reported	Given by study team	Face-to-face	4	60	3.45
Bloos, 2017 [36, 37]	Secondary care	Educational outreach + Educational materials + Remind clinicians	Not reported	Not reported	Given by study team	Face-to-face + Educational materials	6	Not reported	Not reported
Bond, 2011 [38]	Secondary care	Educational meetings + Purposely reexamine implementation + Educational materials + Tailor strategy	Not reported	Not reported	Co-produced	Face-to-face + Virtual (telephone)	9	45	Not reported
Bonevski, 1999 [39]	Primary Care	Educational materials + Provide local technical assistance	Theory of adult behaviour change (Green et al., 1980; Rogers, 1983)	Not reported	Determined by recipients	Face-to-face + Educational materials	2	420	1
Borgiel, 1999 [40]	Primary Care	Educational outreach + Purposely re-examine implementation	Not reported	Not reported	Co-produced	Face-to-face + Virtual (telephone)	3	Not reported	1
Bregnhoj, 2009 [41]	Primary Care	Educational meeting + Educational materials + Purposely re-examine implementation	Not reported	Not reported	Not reported	Virtual (telephone) + Educational materials	3	Not reported	Not reported
Brown, 1994 [42]	Dental	Educational meeting + Educational materials	Adult learning theory (Eisenberg, 1986)	Not reported	Not reported	Face-to-face + Educational materials	3	900	Not reported
Chaillet, 2015 [43]	Secondary care	Educational meeting + Develop and organise quality monitoring system	Not reported	Influence sought by data recipients	Determined by recipients	Not reported	1	450	Not reported
Charrier, 2008 [44]	Secondary care	Facilitation	Not reported	Not reported	Determined by recipients	Face-to-face	5	Not reported	8.6
Clarke, 2020 [45, 46]	Secondary care	Educational meeting + Educational materials + Prepare patients to be active participants + Promote network weaving + Inform local opinion leaders	Instructional design theory (Sinclair et al., 2017)	Influences given	Not reported	Face-to-face + Virtual (online) + Educational materials	1	480	120
Cundill, 2015 [47, 48]	Primary Care	Educational meeting + Educational materials + Promote network weaving + Prepare patients to be active participant + motivational text messages	Programme theory drawing upon different multi-level theories, including Kolb, 1984	Not reported	Determined by recipients	Face-to-face + Virtual (telephone) + Educational materials	3	360	1–5
Curtis, 2011 [49–51]	Secondary care	Educational outreach + Educational meeting + Educational materials + Change equipment	Self-efficacy theory (Bandura, 1982)	Influence sought by data recipients	Co-produced	Face-to-face + Educational materials	3	60	Not reported
DeVore, 2015 [52]	Secondary care	Educational meeting + Educational outreach + Change equipment	Not reported	Not reported	Given by study team	Virtual (telephone + online) + Educational materials	Not reported	Not reported	Not reported
Everett, 1983 [53]	Secondary care	Educational meeting + Educational materials	Not reported	Not reported	Determined by recipients	Face-to-face + Educational materials	5	Not reported	2
Fabbri, 2019 [54]	Primary Care	Network weaving	Unreferenced theory of change ^b^	Influence sought by data recipients	Determined by recipients	Face-to-face	4	Not reported	Not reported
Filardo, 2009 [55–57]	Secondary care	Educational meeting + Educational outreach + Conduct cyclical small tests of change + Identify and prepare champions + Purposely re-examine implementation	Not reported	Not reported	Co-produced	Face-to-face + Virtual (telephone + online)	4	1800	Not reported
Foster, 2007 [58]	Primary Care	Educational meeting + Educational materials + Facilitation + Tailor strategy + Implementation blueprint	Not reported	Influence sought by data recipients	Determined by recipients	Face-to-face + Educational materials	1	180	2
Foy, 2004 [59]	Secondary care	Educational meetings + Educational materials + Tailor strategies + Prepare patients to be active participants	Theory of planned behaviour	Influence sought by data recipients	Determined by recipients	Face-to-face + Educational materials	1	Not reported	Not reported
Frijling, 2002 [60]	Primary Care	Educational outreach + Educational materials + Facilitation + Purposely re-examine implementation	Not reported	Not reported	Co-produced	Face-to-face + Educational materials	15	900	Not reported
Frijling, 2003 [60–67]	Primary Care	Educational outreach + Educational materials + Facilitation	Model of change (Hulscher et al., 1997)	Not reported	Co-produced	Face-to-face + Educational materials	15	900	Not reported
Gilkey, 2014 [68, 69]	Primary Care	Educational outreach + Facilitation + Tailor strategy + Change equipment	Not reported	Influence sought by data recipients	Co-produced	Face-to-face + Virtual (online)	1	90	1
Gilkey, 2019 [70]	Primary Care	Educational meeting + Educational materials	Not reported	Influences given	Not reported	Face-to-face	Not reported	60	Not reported
Gjelstad, 2013 [71, 72]	Primary Care	Educational outreach + Educational meeting + Remind clinicians + Facilitation	Not reported	Not reported	Given by study team	Face-to-face + Educational materials	2	Not reported	Not reported
Guadagnoli, 2000 [73]	Secondary care	Educational outreach + Educational materials	Not reported	Not reported	Not reported	Face-to-face + Educational materials	Not reported	Not reported	Not reported
Gude, 2016 [74, 75]	Secondary care	Educational outreach + Implementation blueprint	Goal setting (Locke and Latham, 2002); Model for improvement (Langley et al., 2009)	Influence sought by data recipients	Determined by recipients	Face-to-face + Virtual (telephone)	5	750	1–11
Gulliford, 2019 [76]	Primary Care	Educational meeting + Educational materials + Change clinical record system + Prepare patients to be active participants	Social cognitive theory (Bandura, 1991) + Self-determination theory (Deci et al., 1980)	Not reported	Not reported	Virtual (online) + Educational materials	1	Not reported	Not reported
Gullion, 1988 [77]	Primary + Secondary Care	Educational meeting + Educational materials	Counselling model with five steps: awareness, motivation, skills, support + maintenance (Green et al., 1979)	Not reported	Not reported	Virtual (telephone) + Educational materials	1	60	Not reported
Harris, 2015 [78]	Primary Care	Educational outreach + Prepare patients to be active participants + Facilitation	Not reported	Influence sought by data recipients	Given by study team	Face-to-face + Educational materials	Not reported	570	Not reported
Hayes, 2001 [79]	Secondary care	Educational outreach + Access new funding	Behavioural change theory (social learning and self-efficacy theories mentioned but not referenced)	Not reported	Determined by recipients	Face-to-face	1	Not reported	1
Hayes, 2002 [80, 81]	Secondary care	Educational meeting + Educational materials + Clinician reminder + Access new funding	Not reported	Not reported	Determined by recipients	Face-to-face	1	Not reported	Not reported
Hendryx, 1998 [82]	Secondary care	Educational outreach + Educational materials + Tailor strategies	Not reported	Co-produced	Given by study team	Face-to-face + Virtual (telephone) + Educational materials	Not reported	Not reported	Not reported
Herbert, 2004 [83]	Primary Care	Educational meeting + Educational materials	Not reported	Not reported	Not reported	Face-to-face + Educational materials	1	120	Not reported
Hogg, 2008 [84, 85]	Primary Care	Educational outreach + Change clinical record system + Facilitation + Tailor strategy	Social learning theory (Unreferenced)	Influence sought by data recipients	Co-produced	Face-to-face + Educational materials	12	552	1–6
Houston, 2015 [86, 87]	Primary Care	Educational outreach + Remind clinicians + Implementation blueprint + Prepare patients to be active participants	Promoting Action on Research Implementation in Health Services (PARIHS) framework	Co-produced	Co-produced	Virtual (online)	7	45	2
Huffman, 2018 [88]	Secondary care	Educational meeting + Educational materials + Conduct cyclical small tests of change + Prepare patients to be active participants	Not reported	Co-produced	Given by study team	Face-to-face + Educational materials	1	120	2
Huis, 2013 [89]	Secondary care	Educational meeting + Clinician reminder + Train for leadership + Model change + Change equipment + Tailor strategy + Local consensus discussion	Social learning theory (Bandura, 1986); Social influence theory (Mittman et al., 1992); Theory on team effectiveness (Shortell et al., 2004; West, 1990); Leadership theory (Øvretveit, 2004)	Influence sought by data recipients	Determined by recipients	Face-to-face + Educational materials	3	270	Not reported
Ivers, 2013 [90, 91]	Primary Care	Implementation blueprint + Formal commitment	Control theory (Carver et al., 1982) Feedback Intervention theory (Kluger et al., 1996); Self efficacy (Bandura, 1994); Theory of planned behaviour (Ajzen et al., 2007; Commitment to change (Shershneva et al. 2010);	Not reported	Determined by recipients	Educational materials	5	Not reported	2.86
Kaufmann-Kolle, 2011 [92]	Primary Care	Form a learning collaborative	Not reported	Not reported	Not reported	Face-to-face	2	Not reported	Not reported
Kennedy, 2015 [93–95]	Secondary care	Educational meetings + Educational materials + Tailor strategy + Implementation blueprint + Conduct cyclical small tests of change	Knowledge to action cycle (Graham)	Co-produced	Determined by recipients	Face-to-face + Educational materials	3	180	5–10
Kiefe, 2001 [96]	Primary + Secondary Care	Implementation blueprint + Prepare patients to be active participants	Theory that comparator data motivates change (Jencks + Wilensky, 1995)	Co-produced	Determined by recipients	Face-to-face + Educational materials	1	Not reported	Not reported
Kritchevsky, 2008 [97, 98]	Secondary care	Form a learning collaborative + Educational meeting + Educational materials	Collaborative learning model	Influence sought by data recipients	Given by study team	Face-to-face + Virtual (telephone)	Not reported	Not reported	Not reported
Lakshminarayan, 2010 [99]	Secondary care	Inform local opinion leaders + Involve executive Boards + Tailor strategies + Educational meeting + Purposely reexamine implementation + Educational materials	Theoretical frameworks of adult learning and behaviour change (Greco et al., 1993)	Influences given	Given by study team	Face-to-face + Virtual (telephone)	Not reported	Not reported	2
Lemelin, 2001 [100, 101]	Primary Care	Facilitation + Local consensus discussion + Inform local opinion leaders + Educational outreach + Remind clinicians + Prepare patients as active participants + Implementation blueprint + Purposely reexamine implementation	Quality improvement framework (Leiniger et al., 1997)	Co-produced	Co-produced	Face-to-face	33	105	Not reported
Lesuis, 2018 [102]	Secondary care	Educational meeting + Change clinical record + Remind clinicians	Not reported	Not reported	Not reported	Face-to-face	1	60	20
Levi, 2020 [103, 104]	Secondary care	Educational meeting + Educational outreach + Educational materials + Purposely reexamine the implementation	behaviour change wheel (Michie et al., 2011)	Co-produced	Determined by recipients	Face-to-face + Virtual (telephone + online)	1	Not reported	2
Lopez-Picazo, 2011 [105]	Primary Care	Educational outreach	Not reported	Not reported	Given by study team	Face-to-face	7	105	Not reported
Lynch, 2016 [106–108]	Secondary care	Educational meeting + Educational materials + Inform local opinion leaders + Tailor strategy	Implementation of change model (Grol et al., 2005)	Influence sought by data recipients	Co-produced	Face-to-face + Educational materials	4	120	Not reported
McCartney, 1997 [109]	Primary Care	Educational meeting	Not reported	Not reported	Not reported	Face-to-face	1	60	Not reported
McClellan, 2004 [110]	Secondary care	Educational meeting + Involve executive boards + Educational materials + Tailor strategy + Purposely re-examine implementation + Outreach	Not reported	Influences given	Co-produced	Face-to-face	1	Not reported	Not reported
McClusky, 2016 [111]	Primary Care	Educational meeting + Educational materials + Tailor strategy + Facilitation	Not reported	Influence sought by data recipients	Determined by recipients	Face-to-face + Educational materials	2	180	Not reported
Mertens, 2015 [112]	Primary Care	Educational meeting + Inform local opinion leaders	Not reported	Influence sought by data recipients	Not reported	Face-to-face	2	150	10.92
Moher, 2001 [113]	Primary Care	Facilitation + Change clinical record system + Educational materials + Formal commitment	Not reported	Not reported	Given by study team	Face-to-face	1	Not reported	Not reported
Mold, 2014 [114]	Primary Care	Educational outreach + Educational materials + Facilitation + Implementation blueprint	Cooperative extension (Rogers, 2003)	Influence sought by data recipients	Determined by recipients	Face-to-face + Educational materials	30	Not reported	Not reported
Mold, 2008 [115]	Primary Care	Educational outreach + Educational materials + Facilitation + Conduct cyclical small tests of change + change clinical record system	Not reported	Not reported	Co-produced	Face-to-face + Educational materials	42	90	Not reported
Myers, 2004 [116, 117]	Primary Care	Educational outreach + Educational materials + Tailor strategy + remind clinicians	Not reported	Influence sought by data recipients	Given by study team	Face-to-face + Educational materials	2	Not reported	Not reported
Nilsson, 2001 [118]	Primary Care	Educational outreach + Educational material	Not reported	Not reported	Not reported	Face-to-face + Educational materials	3	90	Not reported
Palmer, 1985 [119, 120]	Primary Care	Educational meeting + Educational materials + Conduct cyclical small tests of change	Not reported	Not reported	Determined by recipients	Educational materials	4	Not reported	Not reported
Papadakis, 2018 [121, 122]	Primary Care	Facilitation + change clinical record system + remind clinicians + educational meeting + train for leadership	Designed to influence the following 4 factors known to affect self-efficacy: (1) skills targeted clinician self-efficacy through training, (2) personal experience, (3) modelling of behaviours, and (4) positive social or environmental supports (Bandura, 2004)	Influence sought by data recipients	Co-produced	Face-to-face	1	75	Not reported
Patel, 2018 [123]	Secondary care	Remind clinicians + Change clinical record	Not reported	Influence sought by data recipients	Not reported	Face-to-face	3	15	4
Peiris, 2015 [124, 125]	Primary Care	Educational meeting + Change clinical record system	Not reported	Not reported	Not reported	Face-to-face	Not reported	45	Not reported
Pettersson, 2011 [126]	Nursing homes	Educational outreach + Educational materials	Key features of a quality improvement intervention (Hulscher et al. 2003)	Influences given	Determined by recipients	Face-to-face	2	180	2–13
Price-Haywood, 2014 [127, 128]	Primary Care	Clinical supervision + Educational outreach	Not reported	Not reported	Given by study team	Face-to-face	3	30	Not reported
Quanbeck, 2018 [129–131]	Primary Care	Educational materials + Facilitation + Conduct cyclical small tests of change + Educational outreach	Not reported	Influence sought by data recipients	Given by study team	Face-to-face + Virtual (telephone + online)	5	60	8.5
Quinley, 2004 [132]	Primary Care	Educational outreach + Educational materials + Facilitation + Tailor strategy	Not reported	Influence sought by data recipients	Co-produced	Virtual (telephone)	2	Not reported	Not reported
Raasch, 2000 [133, 134]	Primary Care	Educational outreach	Not reported	Influences given	Not reported	Virtual (telephone) + Educational materials	1	Not reported	Not reported
Raja, 2015 [135]	Secondary care	Clinical supervision	Not reported	Not reported	Given by study team	Educational materials	Not reported	Not reported	21–22
Rantz, 2001 [136]	Nursing homes	Educational outreach + Educational materials + Purposely re-examine implementation + Tailor strategy + clinical supervision	Not reported	Influence sought by data recipients	Given by study team	Face-to-face + Virtual (telephone) + Educational materials	Not reported	Not reported	Not reported
Rask, 2001 [137]	Primary Care	Educational materials + facilitation + clinical supervision + change equipment + prepare pts to be active recipients	Not reported	Not reported	Given by study team	Face-to-face	Not reported	Not reported	Not reported
Ruangkanchanasetr, 1993 [138]	Secondary care	clinical supervision + remind clinicians	Not reported	Influences given	Given by study team	Face-to-face	Not reported	Not reported	9
Rubin, 2001 [139]	Secondary care	Educational meeting + Educational materials	Not reported	Not reported	Given by study team	Face-to-face	Not reported	Not reported	Not reported
Sauaia, 2000 [140]	Secondary care	Educational materials + Educational meeting	Not reported	Not reported	Co-produced	Face-to-face + Educational materials	1	120	Not reported
Schectman, 2003 [141]	Primary Care	Educational meeting + Prepare patients to be active participant + educational materials	Not reported	Not reported	Given by study team	Face-to-face + Educational materials	Not reported	90	Not reported
Schneider, 2007 [142]	Primary Care	Form a learning collaborative	Not reported	Not reported	Given by study team	Not reported	Not reported	Not reported	Not reported
Scholes, 2006 [143]	Primary Care	Educational meeting + Educational materials + Remind clinicians + train for leadership	Precede/ proceed planning model (Green et al., 1991)	Not reported	Not reported	Educational materials	Not reported	200	Not reported
Sinclair, 1982 [144]	Secondary care	Educational meeting + clinical supervision	Not reported	Not reported	Given by study team	Face-to-face	9	900	5
Siriwardena, 2002 [145]	Primary Care	Educational outreach + Tailor strategy	Theoretical support from adult learning theory (Miller’s pyramid and competence-performance gap; Miller, 1990)	Influence sought by data recipients	Not reported	Face-to-face	1	60	Not reported
Smith-Bindman, 2020 [146]	Secondary care	Form a learning collaborative + Educational meeting + educational materials	Not reported	Influence sought by data recipients	Co-produced	Virtual (online)	7	630	Not reported
Solomon, 2004 [147]	Secondary care	Educational outreach + Educational materials + Remind clinicians	Not reported	Not reported	Not reported	Face-to-face	3	90	11
Sondergaard, 2005 [148]	Primary Care	Educational meeting + Educational materials + Prepare patients to be active participant	Adult Learning Theories (Fox et al., 1989)	Not reported	Not reported	Face-to-face + Educational materials	2	Not reported	Not reported
Soumerai, 1998 [149]	Secondary care	Educational meeting + Educational materials + Tailor strategy + Inform local opinion leaders + formal commitment	Not reported	Influences given	Determined by recipients	Face-to-face + Educational materials	Not reported	420	Not reported
Stewardson, 2016 [150]	Secondary care	Clinical supervision + educational materials + prepare pts to be active recipients	Not reported	Not reported	Not reported	Face-to-face	Not reported	Not reported	Not reported
Tierney, 1986 [151]	Secondary care	Remind clinicians	Not reported	Not reported	Not reported	Virtual (online)	Not reported	Not reported	135
Trietch, 2017 [152, 153]	Primary Care	Form a learning collaborative + Tailor strategy + Educational materials	Not reported	Influence sought by data recipients	Determined by recipients	Face-to-face	6	120	8–10
Van De Velden, 2016 [154]	Primary Care	Educational outreach + Educational materials + Prepare patients to be active participants	Elements of behavioural change frameworks were included if felt to be useful (Unreferenced)	Not reported	Determined by recipients	Face-to-face + Educational materials	1	90	1–6
van der Weijden, 1999 [155]	Primary Care	Educational meeting + Educational materials + Educational outreach + Tailor strategy + Prepare patients to be active participants	Not reported	Influence sought by data recipients	Given by study team	Face-to-face + Educational materials	3	180	Not reported
Veninga^a^, 1999 [156]	Primary Care	Form a learning collaborative + educational materials	Theories of adult learning (Holm, 1998)	Influences given	Determined by recipients	Face-to-face	2	Not reported	Not reported
Verstappen, 2003 [157]	Primary Care	Educational meeting + Educational materials	Not reported	Influence sought by data recipients	Co-produced	Face-to-face + Educational materials	3	90	6.54
Verstappen, 2004 [157, 158]	Primary Care	Educational meeting + Educational materials + Tailor strategy	Not reported	Influence sought by data recipients	Given by study team	Face-to-face + Educational materials	3	90	Not reported
Vingerhoets, 2001 [159]	Primary Care	Educational materials	Not reported	Not reported	Given by study team	Educational materials	1	Not reported	Not reported
Wahlstrom, 2003 [160]	Secondary care	Educational meeting + Tailor strategy	Not reported	Influence sought by data recipients	Given by study team	Face-to-face	Not reported	90	Not reported
Walsh, 2007 [161]	Secondary care	Educational meeting + Conduct cyclical small tests of change + educational outreach + educational materials	Not reported	Influences given	Determined by recipients	Face-to-face + Virtual (online)	2	Not reported	Not reported
Wang, 2018 [162, 163]	Secondary care	Educational meeting + Educational materials + Tailor strategy	Not reported	Influence sought by data recipients	Determined by recipients	Face-to-face + Educational materials	1	840	2
Wathne, 2018 [164]	Secondary care	Educational outreach + Tailor strategy	Not reported	Influence sought by data recipients	Co-produced	Face-to-face	Not reported	Not reported	Not reported
Widden, 2018 [165]	Primary Care	Clinical supervision + purposively reexamine implementation	Not reported	Influences given	Given by study team	Face-to-face	Not reported	Not reported	Not reported
Willis, 2020 [166–168]	Primary Care	Educational outreach + Tailor strategies + Educational materials + Implementation blueprint + Change clinical record system + Facilitation	Behaviour Change Techniques (Francis et al., 2012); Theoretical Domains Framework (Lawton et al., 2016)	Co-produced	Co-produced	Face-to-face + Educational materials	1	30	Not reported
Wu, 2019 [169, 170]	Secondary care	Involve executive boards + Educational materials + Facilitation + Prepare patients to be active participants	Not reported	Not reported	Given by study team	Virtual (online) + Educational materials	Not reported	Not reported	Not reported

^a^Excluded from Cochrane review due to unclear outcomes

^b^Leveraging providers’ pro-social motivation or by increasing citizens’ voice, community participation, and accountability would encourage participants to devise and implement strategies to improve

**Table 3:** A cumulative summary of the description, content and delivery of included feedback facilitation interventions

**Described…**	**Frequency** (*n* = 104)	**Intervention delivery**	**Frequency** (*n* = 104)
a. using theory	31	k. Modes of delivery	Face-to-face (*n* = 86) Educational materials (*n* = 52) Virtual (telephone) (*n* = 16) Virtual (online) (*n* = 12) Multiple modes (*n* = 58) Unclear (*n* = 2)
b. a logic model	10	l. Frequency of FF	Median = 2 IQR = 1–5
c. identification of priorities	65	m. Duration of FF	Median = 120 min IQR 75-420 min
d. exploration of influences e. determination of implementation strategies	53 80	n. Timing of FF	Before feedback (*n* = 14) With feedback (*n* = 37) After feedback (*n* = 32) Multiple time points (e.g. before and with) (*n* = 10) Unreported (*n* = 32)
f. Identification of implications of performance	43	o. Who delivered FF	Experts (*n* = 50) Peers (*n* = 31) QI specialists (*n* = 21) Computer programme (*n* = 2)
g. tailoring h. that an assessment of fidelity was undertaken	19 41	p. Who received FF	Clinicians (*n* = 86) Clinicians & non-clinical/ managerial (*n* = 10) Unclear (*n* = 8)
		q. Number of recipients per site	Median = 4 IQR = 2–9 Unclear (*n* = 68)
i. the degree of achievement of fidelity	27	r. Number of intervention arm sites	Median = 19 IQR = 12–38 Unclear (*n* = 2)
j. whether the intervention was modified	44	s. Number of people receiving intervention at once	Median = 4 IQR = 1–9 Unclear (*n* = 71)
k. that the intervention was modified	8	t. Level of change sought	Patient (*n* = 2) Team (*n* = 74) Multi-team organisation (*n* = 23)

### Date and setting

**Fig. 2:**
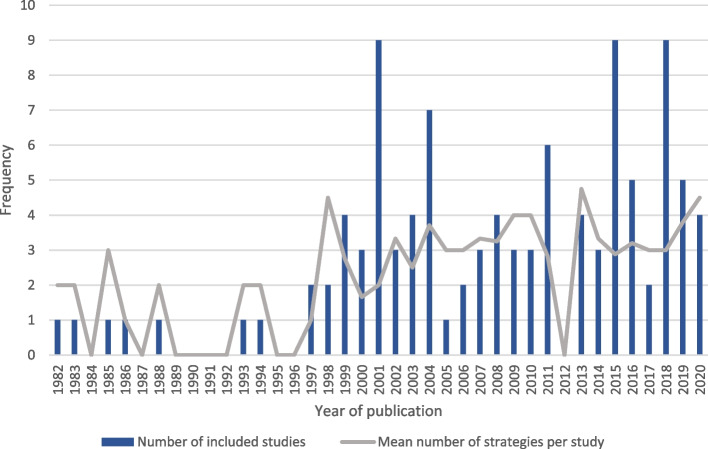
A graph describing the date and frequency of included studies of feedback facilitation and the mean number of strategies used per feedback facilitation intervention

Included trials dated from 1982 to 2020 (Fig. 2). The included studies took place in primary care (*n* = 54; 52%), secondary care (*n* = 43; 41%), two in both primary and secondary care, 3 in nursing homes, 1 in an antenatal clinic (unclear whether primary or secondary care) and 1 in dental practice.

### Expert Recommendations for Implementing Change (ERIC) strategies

**Fig. 3:**
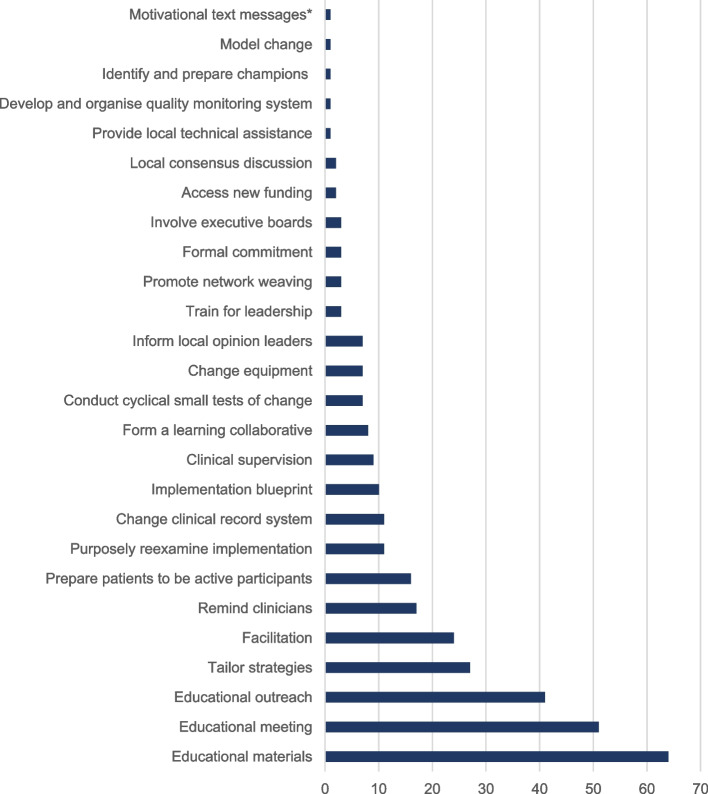
A graph of the frequency of implementation strategy use within included studies [*Added to ERIC coding]

We identified 26 different implementation strategies within feedback facilitation (Fig. 3). The median number of strategies per trial was 3 (IQR 2–4 strategies). Figure 2 shows that the number of strategies used within feedback facilitation interventions has increased over time. There were no apparent differences in the number of strategies used depending on whether the feedback facilitation intervention was undertaken in primary or secondary care (Supplementary materials 6 & 7).

### Use of theory and logic models

We found 35 studies (34%) that described using theory. A total of 31 theories were referenced within the included papers. The most frequent were adult learning theory (*n* = 5; 5%) [e.g. 171], Rogers’ diffusion of innovation theory (*n* = 4; 4%) [172], Bandura’s self-efficacy theory (*n* = 4; 4%) [173], and Bandura’s social learning theory (*n* = 4; 4%) [174]. We found that theory was most frequently used in intervention design. Data from papers published since Colquhoun and colleagues’ exploration of the use of theory in studies of audit and feedback [13] are presented in the Supplementary Materials 2. As illustrated by the quotes, we found that it was often difficult to understand how the authors’ applied theory; for example, “(we) combined strategies shown to change providers’ behaviour with those based on the diffusion of innovation theory” [24] and “technology-assisted learning resources were also developed using motivational systems and instructional design theory” [45].

We found 10 studies provided a logic model to describe the intervention. Table 4 summarises the content of these logic models.

**Table 4:** Components of logic models describing the feedback facilitation interventions

Author	Date	Mechanism(s)		Context	Pre-conditions or Moderator	Outcome(s)	
		**Connection identified**	**Mechanism** **identified**			**Proximal** **Outcomes**	**Distal outcomes**
Ayieko [28]	2011	Yes	Yes, healthcare worker knowledge, values and motivation. Clients resources, knowledge, attitudes and values	Policy and economic environment, provincial and national institutions	Yes, organisational structure, administrative competence, responsiveness, priorities, motivation	No	Yes
Ayieko [30]	2019	Yes	Yes, intrinsic motivation, psychological capability & personal role in improvement	Yes, resource inadequacy, failure of incentives, poor motivation	No	Yes, persuaded about alignment between (forgotten) personal and vocational goals, enhanced self-esteem and peer recognition	Yes, commitment and sense of ownership, but not linked to trial outcome
Cundill [47]	2015	No	Yes, awareness, knowledge, practice and tools to enable change	No	No	Yes, skills, confidence, motivation	Yes
Gilkey [68]	2014	Yes	No	Yes	No	Yes	Yes
Houston [86]	2015	Yes	Some (Staff reminded)	No	No	Yes, knowledge, patient motivation	Yes
Ivers [90]	2013	No	Goal-setting and action-planning	No, although improvement actions tailored to context by participants	Yes, Adherence to instructions, self-efficacy and use of coping plans	Yes, commitment to change	Yes
Lemelin [100]	2001	Yes	No	No	No	Yes, motivation, awareness, attitude change & knowledge	Yes
Lynch [106]	2015	Yes	No – The model describes what is done, not why or how it has an effect	No	No	Yes	No
Quanbeck [129]	2018	No	No	No	No	No	No
Willis [166]	2020	Yes	Yes, both behaviour change technique – determinant couplings and strategy – distal outcome couplings described	Yes, pharmacists and GP contract (Quality Outcomes Framework)	Yes, existing practice meetings	Core were ‘Social & professional role’ and ‘environmental context & resources’. Also prominent were ‘beliefs about consequences’, ‘social influences’, ‘knowledge’ and ‘memory, attention and decision processes’	Yes

### Materials used in feedback facilitation

Feedback facilitation interventions used a range of materials (Supplementary Materials 1). We grouped these into the following categories:

- Materials to support clinician behaviour change by addressing capability, for example, evidence-based guidelines (e.g. [28, 34, 99]), reminder stickers and cards ([e.g. 100, 116, 143]), written educational materials (e.g. [111, 118, 149]). We identified a subset of these materials that was administrative equipment such as patient care record [84], x-ray ordering stamps [93] and ordering sets [99].
- Materials to address clinician motivation; for example, information about reimbursement [132]. It is possible that some of the other materials described above as addressing capability may have addressed motivation (e.g. relating to patient outcome), although this was not clear from the description.
- Materials to support patient behaviour change by addressing capability; for example, patient information leaflets (e.g. [155]) and self-help materials [e.g. 121].
- Clinical equipment to support clinician behaviour change by addressing ‘opportunity’; for example, testing kits (e.g. [24]) and clinical assessment tools (e.g. [114]).
- Materials to support the improvement work: Help to analyse influences (e.g. critical event analysis form [58]; a description of ways to use the audit results, including discussions with colleagues, detailed follow up surveys among patients, and establishment of a patient panel [159]); Help both to select strategies (e.g. written recommendations [27]) and to enact strategies (e.g. action plan [58], amendable template to give information to stakeholders [164]).

### Identification of priorities

We explored whether and by whom priorities were identified from within the performance feedback during feedback facilitation. In 43 studies (43%), priorities for improvement were identified by the feedback facilitators; for example, Hendryx and colleagues’ educational outreach included that “the (study) team member reviewed the findings, and offered concrete, practical suggestions for improvement” ([82] p420). In 19 studies (18%), priorities were identified by feedback recipients; for example, Ivers and colleagues provided a worksheet “to facilitate goal-setting” ([91] p3). In 4 studies (4%), there was evidence that priorities were co-designed between the study team and the feedback recipients [60, 61, 84, 85]. For example, Frijling and colleagues provided feedback facilitation where “the facilitator and the GPs discussed the content of the feedback reports, prioritized specific aspects of decision making to be improved and made change plans” ([60] p837). In 39 studies (38%), it was not possible to determine whether or by whom priorities were identified within the performance feedback.

### Exploration of influences upon performance

We explored whether and how influences upon performance were investigated within feedback facilitation: In 12 studies (12%), influences upon performance were given by the feedback facilitators; for example, “data presented included hospital-specific baseline performance data and information on knowledge and organizational barriers to stroke care identified by the surveys... (including) organisational barriers such as lack of order sets and pathways” ([99] p1635). In 32 studies (31%), influences upon performance were explored by feedback recipients; for example, “a 90-min standardized small group quality improvement meeting, supervised by the medical coordinator of the diagnostic center… (including) a thorough discussion of the difficulties of achieving changes at the individual primary care physician level, the practice level, or at the patient level” ([157] p2408). In 9 studies (9%), we identified that a description of influences upon performance was co-produced by the study team and the feedback recipients. However, there were blurred boundaries between co-produced identification of influences and where influences were sought by feedback recipients; for example, where a focus group might have a facilitator, it was unclear the extent to which they provided a structure or were more directional. In Kennedy et al.’s study, co-ordinators facilitated interdisciplinary care teams to identify “barriers and facilitators to implementing evidence-based strategies, particularly changes that could be made at an organizational level” (p4). In 51 studies, it was not possible to determine whether/how influences upon performance were explored.

Where influences upon performance were given, these may be based upon previous research, including as part of intervention development. Influences upon performance were sought by recipients both in discussion and using proformas. Some focussed on specific barriers (e.g. confidence [78]) whilst others used a broader lens; for example, Chaillet et al. [43] described that, "the training program also sensitized participants to social, economic, organizational, cultural and legal factors”. Proformas were used to support recipients to explore influences (e.g. [58]). The depth of exploration varied (e.g. a 3-h training session [155] or 20-min exercise [111]) and may be a collective (e.g. focus group [27]) or individual exercise (e.g. [116]). Co-production included national analysis followed by local tailoring, information gathering from patients followed by healthcare worker selection and the sharing of learning between sites.

There were no apparent differences in whether the influences were sought by recipients, given or co-produced depending on whether the feedback facilitation intervention was undertaken in primary or secondary care (Supplementary materials 7).

### Determining implementation strategies

We explored how strategies were selected: In 33 studies (32%), improvement strategies were given by the feedback facilitators, in 27 studies (26%) they were determined by the feedback recipients. Improvement strategies were co-designed in 20 studies (19%). In 24 studies (23%), it was not reported who determined the improvement strategies.

The suggested strategies given by the study team were sometimes generic suggestions to all teams (e.g. [52]), and sometimes site specific (e.g. [34]). Where the strategies were determined by recipients, this included doing so with the support of learning from other sites [126] and using a plan-do-study-act template [93]. Co-produced strategy selection included selection from a list of strategies provided by the study team and adaptation of proposed strategies (e.g. [68]). Proposed strategies could be in a list presented by peers (e.g. [47]), and/or described in meeting, webinars or calls (e.g. [146]).

The Sankey chart (Supplementary Materials 3) illustrates the lack of relationship between who identified influences and who identified strategies: In 10 (10%) trials, both the identification of influences and identification of strategies was undertaken by recipients; in 4 (4%) trials, both were given by the study team.

There were no apparent differences in whether the actions were determined by recipients, given or co-produced depending on whether the feedback facilitation intervention was undertaken in primary or secondary care (Supplementary materials 7).

### Identification of implications of performance

We explored whether feedback facilitation involved identifying implications of performance. We found that implications were given as part of feedback facilitation in 36 studies and identified by feedback recipients in 7 studies (7%). In 61 studies (59%), consideration of implications was not reported. There were no apparent differences in whether the feedback facilitation intervention was undertaken in primary or secondary care (Supplementary materials 7).

### Other intervention components

We looked for additional components to the intervention, not described above. We found additional components that sought to address capability and motivation: Components to address capability targeted both capability to improve (e.g. [68]) and capability to deliver care (e.g. [89]). Interventions to increase motivation included motivational text messages (e.g. [47]), celebrating good practice (e.g. [82]), and positional leader prioritisation [e.g. [82]. These may have had some impact upon the social opportunity by changing the social environment (e.g. giving permission). We did not identify additional components that specifically targeted ‘opportunity’ for the target behaviours, defined as factors that lie outside the individual that make the care or improvement behaviour possible.

### Delivery of feedback facilitation

A variety of modes were used to deliver facilitation, with the most common being face-to-face (*n* = 86; 83%) and educational materials (*n* = 52; 50%). Virtual delivery by telephone (*n* = 16;15%) and online (*n* = 12; 12%) was less commonly used, which is likely to be due in-part to the age of the literature. Most studies used one (*n* = 45; 43%) or two (*n* = 50; 48%) methods of delivery, with fewer using three (*n* = 7; 7%; [38, 45, 47, 52, 82, 84, 136]).

### Frequency of feedback facilitation

Most studies delivered feedback facilitation between 1 and 3 times (median = 3, interquartile range 1–5). Six studies (6%) delivered facilitation 15 times or more [24, 60, 61, 100, 114, 115]). The maximum times feedback facilitation was delivered was 42 times [115]. Data was not available for 25 studies (24%).

### Duration of feedback facilitation

Feedback facilitation delivery took between 15–1800 min, with a median of 120 min and IQR of 75–420 min. For studies with over 420 min of facilitation, this was delivered over several consecutive days and/or as follow up calls following initial delivery. 45 studies (43%) did not record the delivery time.

### Timing of feedback facilitation

Most facilitation was delivered with (*n* = 37; 36%) or after (*n* = 32; 31%) feedback delivery, so that the feedback could be reviewed with the participants. There were some studies that delivered before (*n* = 14; 13%), although ten of these studies (10%) also included facilitation during and/or after feedback. The five studies (5%) that only delivered facilitation pre-feedback all included educational materials. Three of these studies (3%) reported local identification of priorities [39, 42, 58], whilst it was not reported in the other two [43, 76].

### Who delivered feedback facilitation

Most facilitation was delivered by experts (e.g.specialist physicians with expertise in osteoporosis or geriatrics [93]) (*n* = 50; 48%), followed by peers (e.g. local co-ordinators [24] (*n* = 31; 30%) and then quality improvement specialists (*n* = 21; 20%)) (Supplementary Materials 4). Facilitation was delivered virtually through a computer programme in two studies (2%) [39, 151] (Supplementary Materials 1). We discuss challenges with coding this data below.

### Who received feedback facilitation

The majority of facilitation was delivered to clinicians (*n* = 86; 83%), with a smaller number including both clinicians and non-clinical/managerial (*n* = 10; 10%). There were no instances of facilitation being delivered to managers only. In eight studies (8%), it was unclear who were the recipients.

### Number of recipients receiving feedback facilitation per site

It was difficult to determine the number of recipients of facilitation per site, with 68 studies (65%) either not reporting or providing unclear descriptions. The number of recipients per site ranged from 1 to 135. Studies variably described both minimum and maximum recipients per site, with others giving averages but no range. Of the 36 studies (35%) reporting recipients per site, most sites had small groups of 10 or fewer recipients (*n* = 28; 28%).

### Number of intervention sites receiving feedback facilitation

The number of intervention sites ranged from 1 to 811, with a median of 19 and IQR of 12–38. Data was skewed to the right by 18 studies with large intervention site numbers over 50. Two studies (2%) did not report the number of intervention sites [123, 127].

### Comparison of recipients per site with number of intervention arm sites

Where both number of recipients per site and number of intervention sites were recorded (*n* = 33; 32%), the trend was for the number of recipients per site to decrease as the number of intervention sites increased, however this was not statistically significant on linear regression (*p* = 0.86, Confidence intervals –0.55 to 0.21) (Supplementary Materials 5).

### Number of people receiving the intervention at one time

Most studies (*N* = 71; 70%) did not record the number of people receiving the intervention at each time; Of the 33 studies (32%) that did, the intervention was delivered most frequently to an individual (*n* = 9; 9%) and most were delivered to 10 or fewer individuals (*n* = 28; 27%). Two studies (2%) [98, 102] delivered to 11–20 people and three (3%) to 21 or more [70, 147, 162]. The maximum number of people the intervention was delivered to at one time was 45 [70].

### Comparison of number of people receiving the intervention at one time by setting

Interventions delivered in secondary care were often delivered to a larger number of people than those delivered in primary care (secondary care median = 8, IQR = 2.5–16; primary care median = 3.5, IQR = 1–7.5), however this was not a statistically significant finding on a Mann–Whitney U test (*p* = 0.16) (Supplementary materials 6). This is likely due to primary care studies involving feedback to individual practitioners and smaller team sizes compared to secondary care. The lack of studies describing how many people received the intervention at one time makes drawing conclusions difficult.

### Level of change sought

Most facilitation sought a level of change at the team level (*n* = 74, 71%), with fewer studies seeking level of change at multi-team organisation levels (*n* = 23, 22%), at the wider system (*n* = 5, 5%), 2 studies directly targeted patient-level change [45, 150]. For example, Clarke and colleagues provided evidence-based education for women through two antenatal classes as part of an intervention to increase the rates of vaginal birth after caesarean section.

### Tailoring of feedback facilitation

Only 19 studies (19%) reported tailoring of the intervention delivery. Types of tailoring included tailoring of the content to identified needs and barriers and local context (e.g. [34, 73, 122, 161, 166]) and additional episodes of facilitation in response to need (40,131). For example, Quinley and colleagues focussed facilitation on physicians with poorer performance where a practice contained multiple physicians [132]. Brown and colleagues [42] tailored content to existing level of knowledge and delivery through, “the use of a variety of media including individualised tuition and feedback” (p443).

### Assessment of fidelity

Fidelity of facilitation was reported and described as assessed in 41 studies (39%). Where assessed, 27 out of the 41 studies reported fidelity achievement, given either as a range or mean adherence. Fidelity ranged from 29 to 100%.

### Modification

Most studies (*n* = 60, 58%) did not report whether any modifications to the intervention took place. Of those that did, 18% (*n* = 8) reported making modifications whereas 82% (*n* = 36) did not. Examples of modifications included additional re-training sessions [24], modifications due to online system malfunctions [38], changes to number of facilitation sessions offered [74] and delivery mode, for example, where the source was unable to continue to deliver feedback facilitation in-person, so later delivery changed to teleconference  [93]. Reporting of the presence or not of modifications to facilitation interventions is improving over time, with 53% of studies reporting modifications published in 2010 or later and 88% since 2000.

### Reporting of TIDieR intervention content items

The number of TIDieR items not reported within each study was determined to give a score out of 18. The results are presented in Supplementary Table 1b. The non-reporting of items ranged from 2 to 14, with a median of six content items not recorded (IQR 4.75–8). The number of items not reported reduced over time (*p* < 0.05) on linear regression, however this only explained 5% of the variation. Heteroskedasticity was not present on testing (*p* = 0.72).

## Discussion

We describe the content and delivery of the feedback facilitation to support designers of future feedback facilitation interventions. Our systematic review of 146 papers describes feedback facilitation delivered alongside audit and feedback in 104 randomised controlled trials. The papers were identified during the Cochrane review of audit and feedback. [21] The Cochrane review includes an assessment of the effectiveness of feedback facilitation.

We found feedback facilitation is a heterogeneous intervention containing at least one of 26 different implementation strategies and drawing upon each of the 9 implementation strategy groupings [175]. We found evidence that the number of strategies used per intervention is increasing over time (Fig. 2). To support future delivery of feedback facilitation we have used this heterogeneity to illuminate previous intervention design choices (Table 5). This is not intended to represent an exhaustive list of choices. In making these choices, guidance [e.g. 14] recommends intervention developers draw upon evidence, theory and stakeholder views about patient outcomes, proximal outcomes, mechanisms, context, pre-conditions and/or moderators and the intervention content. Articulating these may both support consideration of the coherence of the intervention and evaluation of whether it was provided as planned. Detailed description of planned and actual content also supports learning and replication of delivery. We propose both future work with stakeholders to evolve the design options, and further studies evaluating the impact of these choices upon effectiveness and implementation outcomes such as feasibility, appropriateness and acceptability [176].

**Table 5:** Design options within feedback facilitation v1 (Non-exhaustive)

Design question	Feedback Facilitation exemplar design choices
Who will identify improvement priorities?	Previous feedback facilitation providers: • Identified areas for improvement • Asked feedback recipients to identify areas for improvement • Co-produced improvement priorities
Who do you want to do what differently?	Previous feedback facilitation providers targeted: • Clinicians to provide different care ± • Clinicians to undertake implementation activities ± • Other healthcare workers to undertake implementation activities ± • Patients and carers to access different care ± • Patients and carers to undertake implementation activities (e.g. ask clinicians for a treatment)
How will you identify influences upon current care / audit performance?	Previous feedback facilitation providers: • Undertook research to identify national / local influences upon practice ± • Gave feedback recipients information about identified influences, which they may/may not select from ± • Gave feedback recipients tools to identify local influences ± • Gave feedback recipients the opportunity to discuss influences with other feedback recipients • Co-produced influences upon care • Gave no support to identify influences
How will feedback recipients select improvement actions?	Previous feedback facilitation providers: • Gave feedback recipients a verbal or written list of potential actions ± • Gave feedback recipients the opportunity to discuss actions with other feedback recipients • Co-produced improvement actions • Gave no support to identify influences
Will feedback recipients monitor improvement?	Previous feedback facilitation providers that supported monitoring, did so: • Individually • Collectively within their team • Collectively with other feedback recipients
Will you provide support materials for feedback recipients to improve?	Previous feedback facilitation providers gave materials that: • Supported motivation to improve (e.g. by giving information about consequences) • Supported capability to improve (e.g. training in improvement, materials like action plans to help recipients to improve) • Supported capability to provide/receive best care (e.g. training in the clinical condition, providing guidelines, provide patient self-help or information materials) • Supported opportunity to provide care (e.g. providing testing kits or assessment tools)
How will you implement the above work in feedback recipients?	Previous feedback facilitation providers used 26 different implementation strategies, as described in Fig. 3. Each of these have different active ingredients^a^ Delivery of the intervention may vary in: • The extent to which it is virtual ± face-to-face ± delivered in materials • How often each participant takes part (e.g. once only, monthly) • How many participants take part per site • Duration in total and per contact • The extent to which it is before/with/after feedback • Who delivers the intervention (e.g. clinical expert, peer, quality improvement facilitator) • How many sites receive the intervention at once and in total
Will all sites receive the same?	Previous feedback facilitation providers: • Tailored delivery to the needs to different sites • Gave all sites the same

^a^Guidance recommends that the connections between patient outcomes, proximal outcomes, mechanisms, context, pre-conditions and/or moderators should be described in an illustrative logic model and narratively in greater detail

### These design choices have important implications, including those related to tailoring and dose

In relation to tailoring, the source of both the influences and the selection of improvement actions may impact upon the effectiveness of the intervention; for example, strategies selected by the study team may have a more explicit link to theory and evidence, and may include external stakeholders able to challenge existing mental models. Conversely, the study team’s interpretation of the influences upon performance and the alignment between influences and strategies may differ from those involved in change-making, which might undermine buy-in and create barriers to specification of the change. Future research that investigates the impact of different sources and of co-produced tailoring would support providers of feedback facilitation.

We measured the ‘dose’ of the facilitation and found wide variation, including the duration (15 to 1800 min), frequency of facilitation (1 to 42 episodes) and the number of recipients per site (1 to 135). There was also wide variation in the number of people receiving the intervention at once (1 to 45) and different modes used (e.g. through materials, face-to-face or virtual approaches). Future work could investigate the most (cost-) effective way to deliver feedback facilitation; for example, through the use of SMART optimisation designs [177] with economic evaluations. Such studies should assess both cost of delivery and of receipt. Consideration of real-life scalability would be valuable, given only one study delivered to more than 150 sites. All studies delivered facilitation to an intervention group. Questions remain about whether an adaptive intervention delivering sequences of feedback facilitation strategies as a co-intervention to audit and feedback, where the type, intensity or modality of the co-intervention evolve according to changing recipient responsiveness to feedback, might be more (cost-) effective.

Implementation strategies may contain different behaviour change techniques and act upon different mechanisms [17, 178]. The heterogeneity of feedback facilitation undermines the ability to draw conclusions about its effectiveness. We found that the ERIC compilation provided a valuable tool for identifying component strategies. However, given more recent work describing potential behaviour change techniques within strategies [178], it would support replication and learning if future papers describe the active ingredients (such as, instruction on how to perform behaviour, information about health consequences or social support) within strategies. We identified overlap in the content of ERIC strategies; for example, learning collaboratives often contained educational meetings, re-examining implementation, small tests of change, whilst other studies that also delivered these elements to multiple sites at once may not be described as a learning collaborative. Where an intervention was in the overlap between ERIC definitions, we used the terms used by the authors to categorise the intervention components. We were unable to code motivational text messages [47] using ERIC and included them as an additional strategy. Similarly, we determined that ‘clinical decision support systems’ incorporated both ‘change the clinical record system’ and ‘remind clinicians’ as the closest match. We found that 47 ERIC strategies were not incorporated into feedback facilitation (Supplementary materials 8) and may provide alternative content to future feedback facilitation providers; for example, to promote adaptability.

We explored whether reporting was improving over time. We found that later reported studies had fewer non-reports of TIDieR items as expected with changes in publishing requirements, but this only explains 5% of the variance. Further action to improve reporting may be needed to support interpretation of results, replication of interventions and the advancement of implementation science. We draw particular attention to the omission of the rationale and proximal target of the intended change.

As recommended in TIDieR [11], we sought the underlying rationale for the use of feedback facilitation: 35 studies referenced the use of theory and 10 studies provided a logic model describing their programme theory. Understanding the underlying rationale for an intervention supports replication, as adaptation around core components increases fit to the new context [14]. Describing the programme theory of an intervention also supports interpretation of results; for example, consideration of the coherence of the intervention, the proposed mechanism of effect, the context, the work being done by the intervention recipient and the assessed outcomes. Detailing causal pathways helps advance implementation science [17]. We found that within the 10 trials that had a logic model, there were gaps in the reporting of mechanisms (reported in 6 studies) and of contextual, predisposing or moderating factors (reported in 5 studies); Studies reporting this detail dated from 2011.

We found that feedback facilitation interventions sought to address motivation and capability. This was evidenced within: the proximal and distal outcomes where logic models were provided; the intervention materials (e.g. providing guidelines, detailing impacts upon outcomes and information about reimbursement and providing patient information and self-help materials); and the additional components. In relation to capabilities, the interventions sought to target both capabilities to improve (e.g. support to analyse influences upon care using a critical event analysis form or action plan template) and capabilities to deliver care (e.g. reminder cards or guideline documents). However, the target of the intended proximal change was often unclear; for example, whether education targeted improvement capabilities or knowledge about clinical care. Behaviour change literature (e.g. [179]) recommends specifying the target behaviour prior to the development of interventions. Omitting this information again hampers replication and the advancement of knowledge about what influences different behaviours. We found few examples of interventions addressing opportunity. Interventions may be enhanced by supporting the opportunity to undertake the improvement work; for example, by explicitly bringing that work into a workshop [9].

### Strengths and limitations

There were minor variations from the protocol: We had planned to exclude papers that provided training in the target care practice, rather than the use of feedback, but found that it was not possible to identify the target behaviour of such training. We also planned to explore the extent to which the co-intervention was solely feedback facilitation but heterogeneity within feedback facilitation undermined our ability to assess this.

We sought the presence of a logic model, as recommended by guidance [19]. More recent guidance [14] recommends that a logic model is accompanied by a more detailed description of the programme theory; some studies (e.g. [123]) provided a narrative summary of the programme theory without a logic model. We included 146 papers describing 104 trials, however as with all reviews, there is a risk that we missed papers. We focussed on feedback facilitation within trials, which may differ from feedback facilitation undertaken outside of clinical trials. We included one paper [156] which described feedback facilitation alongside audit, but that was subsequently excluded from the Cochrane review due to the nature of the outcomes measured. Our data extraction template was adapted from the TIDieR framework, with the addition of prompts to seek strategy type, whether/how priorities for improvement were identified, whether/how influences upon performance were sought, whether/how strategies were selected and whether/how implications from performance were identified. Whilst we also sought other components to the intervention, it is possible that different prompts may have identified alternative factors important to the design and delivery of feedback facilitation. It is possible that increased granularity by categorising at the level of behaviour change technique (BCT) rather than ERIC strategy may have been useful, however gaps in recording would have been amplified at the active ingredient level. It is also possible that future feedback facilitation reviewers are seeking information about the mode of delivery found in ERIC strategies but missing from BCTs. We resolved disagreements through discussion but did not keep a record of the content of the discussion. The intervention deliverer (e.g. expert, peer) was difficult to assess from the information provided. It is possible that what is key is whether they are perceived as 'experts' or 'peers' (for example, if they are viewed as ‘credible source’ [180]), an assessment which might be made by each participant, rather than on the basis of a job title. In piloting, the reviewers found it difficult to agree on whether strategies addressed capability, opportunity or motivation. As a result, this was assessed by two reviewers (MS and SA) with training and experience of using COM-B [15] as part of a focussed assessment of the target of specific strategies. We focussed on the content and delivery and characteristics of the feedback recipients, the setting and the level of change sought. We did not collect information about the target behaviours upon which feedback is being given. Further work to explore the relationship between characteristics of the target behaviour(s) and the content and delivery of feedback facilitation may identify additional design choices.

## Conclusion

Feedback facilitation is a much-used intervention delivered alongside large-scale audit and feedback to increase effectiveness. Health system policy and theory-informed hypotheses advocate for the delivery of feedback facilitation, often referred to as support for quality improvement. We describe heterogeneity in the design of feedback facilitation, highlighting some of the design choices for future providers (Table 5). We were able to describe the components with feedback facilitation using ERIC, but there was the opportunity for some minor clarifications in terms and for intervention providers to provide greater specificity. Whilst reporting demonstrated extensive gaps, hindering replication and learning, there was some evidence that reporting is improving over time. We recommend future work to consider the role of ‘opportunity’ within intervention designs and the use of evaluation techniques to maximise intervention efficiency.

### Supplementary Information

Supplementary Material 1.

Supplementary Material 2.

## Data Availability

All data generated or analysed during this study are included in this published article [and its supplementary materials].

## Abbreviations

A&F: Audit and facilitation
BCT: Behaviour change techniques
ERIC: Expert Recommendations for Implementing Change
FF: Feedback facilitation
HQIP: Health Quality Improvement Partnership
IQR: Inter-quartile range
PRISMA: Preferred Reporting Items for Systematic Reviews & Meta-Analyses (Appendix B)
QIC: Quality Improvement Collaborative
SMART: Sequential Multiple Assignment Randomized Trial
TDF: Theoretical domains framework
TIDieR: Template for Intervention Description and Replication
