# Supplementary material for: Exploring the content and delivery of feedback facilitation co-interventions: a systematic review
Source: Implement Sci. 2024 May 28;19:37. doi: 10.1186/s13012-024-01365-9 (PMC11134935; doi:10.1186/s13012-024-01365-9)
Supplement: Supplementary file 1 — Supplementary Material 1. [file 13012_2024_1365_MOESM1_ESM.docx]

**Supplementary Table 1:** A summary of the content and delivery of the included feedback facilitation interventions

| **Trial paper first author + year** | **Materials to support use of feedback** | **Identification of priorities from data** | **Info on implications of performance** | **Other intervention components were identified:** | **How delivered** | **Timing in relation to feedback** | **Who delivered the intervention** | **Who received the intervention** |
| --- | --- | --- | --- | --- | --- | --- | --- | --- |
| Althabe, 2019 | Reminder materials and syphilis testing kits | Not reported | Not reported | Not reported | Face-to-face | Before & With | Peers | Clinicians |
| Avery, 2010 | Log book | Given | Implications given | Not reported | Face-to-face | After | Peers | Clinicians |
| Awad, 2006 | Written specific recommendations | Given | Not reported | Not reported | Face-to-face + Educational materials | With & After | Experts & Peers | Clinicians |
| Ayieko, 2011 | Evidence-based guidelines and job aides (e.g. charts, forms) | Not reported | Not reported | Not reported | Face-to-face + Educational materials | With | Experts & Peers | Clinicians |
| Ayieko, 2019 | Action plan and protocol booklet | Given | Not reported | Not reported | Face-to-face | Not reported | Experts | Clinicians |
| Baker, 1997 | Reminder cards | Given | Implications given | Not reported | Face-to-face + Educational materials | Not reported | Experts | Clinicians |
| Baldwin, 2010 | PowerPoint and DVD presentations | Given | Not reported | Not reported | Face-to-face + Educational materials | After | Peers | Clinicians |
| Barkun, 2013 | Guidelines and algorithm | Not reported | Implications given | Not reported | Face-to-face | With | Experts | Clinicians |
| Bertoni, 2009 | Personal digital assistant, printer adapter paper copies of guidelines | Not reported | Not reported | Recipients who rarely used the PDA with the clinical decision support system were encouraged to do so. | Face-to-face | Before & After | Experts | Clinicians |
| Bloos, 2017 | Active reminders (monthly emails) and passive reminders (pocket cards, flyers, posters) | Given | Implications given | Not reported | Face-to-face + Educational materials | Not reported | Experts | Clinicians |
| Bond, 2011 | Printed materials and online resources | Given | Explored by data recipients | Not reported | Face-to-face + Virtual (telephone) | Not reported | Experts & QI Specialist | Not reported |
| Bonevski, 1999 | Computerised 'continuing medical education' package | Supports local identification | Not reported | Not reported | Face-to-face + Educational materials | Before | Virtual | Clinicians |
| Borgiel, 1999 | Continuing medical education plan and follow up letter summarizing outreach visit | Given | Not reported | Not reported | Face-to-face + Virtual (telephone) | After | Peers | Clinicians |
| Bregnhoj, 2009 | Written recommendation targeting medical problems | Not reported | Implications given | Not reported | Virtual (telephone) + Educational materials | Before & After | Peers | Clinicians |
| Brown, 1994 | Bimonthly newsletters | Supports local identification | Not reported | Not reported | Face-to-face + Educational materials | Before | Peers | Clinicians |
| Chaillet, 2015 | Clinical algorithms and recommendations for best practices | Not reported | Not reported | Not reported | Not reported | Before | Peers | Clinicians |
| Charrier, 2008 | Checklist | Not reported | Not reported | Not reported | Face-to-face | With | QI Specialist | Clinicians |
| Clarke, 2020 | Learning resources | Given | Not reported | Not reported | Face-to-face + Virtual (online) + Educational materials | Not reported | Peers | Clinicians |
| Cundill, 2015 | Clinic posters and leaflets | Not reported | Implications given | Motivational text messages | Face-to-face + Virtual (telephone) + Educational materials | After | Peers | Clinicians |
| Curtis, 2011 | Video presentations and pamphlets | Not reported | Implications given | Not reported | Face-to-face + Educational materials | With | Not reported | Clinicians |
| DeVore, 2015 | Specialised tool kits (e.g. patient instructions and order set templates) | Not reported | Not reported | The lowest performers (bottom 25%) were also targeted with additional phone calls and webinars to develop solutions for improvement. Specialized tool kits were administered to all intervention sites and provided resources, such as patient instructions and order set templates. | Virtual (telephone + online) + Educational materials | Not reported | Experts & Peers | Not reported |
| Everett, 1983 | Cost-education newsletters and charge profiles for common lab tests | Supports local identification | Implications given | Not reported | Face-to-face + Educational materials | Not reported | Experts | Clinicians |
| Fabbri, 2019 | Not reported | Not reported | Implications given | Not reported | Face-to-face | With | Experts | Clinicians |
| Filardo, 2009 | Educational curriculum reported | Supports local identification | Not reported | Not reported | Face-to-face + Virtual (telephone + online) | With | QI Specialist | Clinicians & Managers |
| Foster, 2007 | Critical event analysis form, references, resources (e.g. guideline summary charts, web-sites of professional organisations, training organisations, equipment manufacturers) and examples of asthma action plans | Supports local identification | Not reported | Not reported | Face-to-face + Educational materials | Before | Peers | Clinicians & Managers |
| Foy, 2004 | Patient information booklet and supportive evidence | Given | Not reported | Not reported | Face-to-face + Educational materials | With | Experts | Not reported |
| Frijling, 2002 | Unspecified educational materials | Co-designed | Not reported | Not reported | Face-to-face + Educational materials | After | QI Specialist | Clinicians |
| Frijling, 2003 | Not reported | Co-designed | Not reported | Not reported | Face-to-face + Educational materials | After | QI Specialist | Clinicians |
| Gilkey, 2014 | Adobe screen sharing software for virtual facilitation | Supports local identification | Not reported | Promoted information exchange and acknowledge and reward improved performance during later visit | Face-to-face + Virtual (online) | With | Experts | Clinicians |
| Gilkey, 2019 | Powerpoint presentation ,video vignettes and educational materials | Given | Implications given | Not reported | Face-to-face | Not reported | Peers | Clinicians |
| Gjelstad, 2013 | Some participants also received pop-up reminders asking about whether it was a delayed prescription | Given | Explored by data recipients | Not reported | Face-to-face + Educational materials | Before & After | Peers | Clinicians |
| Guadagnoli, 2000 | Presentations and graphic materials | Not reported | Not reported | Not reported | Face-to-face + Educational materials | Not reported | Peers | Not reported |
| Gude, 2016 | Template (through online portal) | Supports local identification | Not reported | Not reported | Face-to-face + Virtual (telephone) | After | QI Specialist | Clinicians |
| Gulliford, 2019 | Patient information sheets and advice on the indications for antibiotic prescription. Computer-aided decision support tool. | Not reported | Implications given | Not reported | Virtual (online) + Educational materials | Before | Peers | Clinicians |
| Gullion, 1988 | Peer-reviewed syllabus material | Not reported | Not reported | Not reported | Virtual (telephone) + Educational materials | With | Experts & Peers | Clinicians |
| Harris, 2015 | Patient education resources | Supports local identification | Implications given | Not reported | Face-to-face + Educational materials | Not reported | QI Specialist | Clinicians |
| Hayes, 2001 | Educational slide show, video and quality improvement tools | Not reported | Not reported | Aim for physicians to increase commitment through social support and act as a role model. Physician liaisons were paid a $500 honorarium on submission of their hospital's QI plan. | Face-to-face | With | Peers & QI Specialist | Clinicians |
| Hayes, 2002 | An educational slide, an example of a chart reminder system to reinforce target dosing, a monthly newsletter and a protocol designed to improve echocardiography reporting | Not reported | Not reported | Physician liaisons were paid a $500 honorarium on submission of their hospital's QI plan | Face-to-face | With | Peers | Clinicians |
| Hendryx, 1998 | Quarterly newsletters, reprints of articles and description of relevant university-based seminars. | Given | Implications given | Areas of good practice celebrated to establish trust and increase willingness to address areas for improvement. Verbal feedback to clinical team, followed by written feedback to positional leaders. Telephone helpline to consult on specific patients, equipment and protocol questions. | Face-to-face + Virtual (telephone) + Educational materials | After | Peers | Clinicians |
| Herbert, 2004 | 8-page module | Not reported | Implications given | Not reported | Face-to-face + Educational materials | With | Not reported | Clinicians |
| Hogg, 2008 | Prevention flow sheets, chart flags, sticker reminders, electronic reminders and patient care record | Co-designed | Not reported | Not reported | Face-to-face + Educational materials | With | Peers | Clinicians |
| Houston, 2015 | Pre-printed pads of “information prescriptions” and the smoking cessation website address. | Not reported | Not reported | Not reported | Virtual (online) | Not reported | Peers | Clinicians & Managers |
| Huffman, 2018 | PDSA templates, standardised admission and discharge order sets, patient education materials on smoking cessation, dietary advice and physical activity, linkage to emergency care cardiovascular training, and online health care quality and patient safety training | Supports local identification | Not reported | Not reported | Face-to-face + Educational materials | Not reported | Experts & QI Specialist | Not reported |
| Huis, 2013 | Written information about hand hygiene | Not reported | Not reported | Setting norms and targets within the team involved three interactive team sessions (1–1.5 h each) guided by the team manager and an external coach. Sought to instigate nurses addressing each other in case of undesirable hand hygiene behaviour and gain commitment. Ward manager designated hand hygiene as a priority and discussed hand hygiene compliance rates with team members. Modelling of good hand hygiene behaviour and how to address behaviour of colleagues by informal leaders. | Face-to-face + Educational materials | With | Peers & QI Specialist | Clinicians & Managers |
| Ivers, 2013 | Explanatory document describing data collection, recommendations, generic quality improvement strategies (e.g. work with administrative staff to encourage patients to have periodic visits), and two-page self-reflection survey. | Supports local identification | Not reported | Not reported | Educational materials | With | Experts | Clinicians |
| Kaufmann-Kolle, 2011 | Not reported | Given | Not reported | Not reported | Face-to-face | After | Not reported | Clinicians |
| Kennedy, 2015 | Falls prevention toolkits (posters, DVD, panel cards), action planning worksheet, case-study exercise, website overview, process checklists, paper-based treatment alerts to assist pharmacy flagging high-risk pts, x-ray requisition stamps, pocket cards, website with interactive forum, and on-going support via website forum | Not reported | Not reported | Not reported | Face-to-face + Educational materials | With | Experts | Clinicians & Managers |
| Kiefe, 2001 | Improvement plan template and patient educational materials | Not reported | Not reported | Not reported | Face-to-face + Educational materials | With | QI Specialist | Clinicians |
| Kritchevsky, 2008 | Presentation given but materials not clear | Supports local identification | Not reported | Not reported | Face-to-face + Virtual (telephone) | After | Experts | Clinicians |
| Lakshminarayan, 2010 | Staff surveys to identify key knowledge and skill deficits and other barriers to stroke care. order sets, pathways, links to practice guidelines, relevant articles, and copies of tools (e.g., patient education tool) | Given | Implications given | Recipients asked to present feedback to specified professional groups | Face-to-face + Virtual (telephone) | Not reported | QI Specialist | Clinicians |
| Lemelin, 2001 | Educational materials, reminder system and patient education materials. | Co-designed | Not reported | The co-intervention sought to: facilitate the development of a practice policy for preventive care, assist in setting goals and desirable levels of performance, facilitate development of a written improvement plan, assist in the development and adaptation of tools and the strategies to improve, facilitate meetings to assess progress and modify the plan if necessary. | Face-to-face | With | Experts | Clinicians |
| Lesuis, 2018 | PowerPoint slides | Not reported | Implications given | Computerized decision support system | Face-to-face | With | Experts | Clinicians |
| Levi, 2020 | Web-based training modules | Supports local identification | Not reported | Multiple forms of educational meeting: preworkshop meetings, collaborative communal workshops, site-based working groups and bimonthly inter-site teleconference | Face-to-face + Virtual (telephone + online) | Before, With & After | Experts & Peers | Clinicians |
| Lopez-Picazo, 2011 | Not reported | Given | Implications given | Not reported | Face-to-face | With | Peers | Clinicians |
| Lynch, 2016 | Minutes of strategy development session, proposed actions | Given | Implications given | Not reported | Face-to-face + Educational materials | With | Experts | Clinicians |
| McCartney, 1997 | Not reported | Not reported | Not reported | Not reported | Face-to-face | With | Experts | Clinicians |
| McClellan, 2004 | Guidelines as well as resources to assist facility staff in conducting a CQI project to improve hemodialysis adequacy (6–7). The materials for patients included videos, booklets, and brochures related to the NKF-DOQI Guidelines and the importance of receiving adequate dialysis. | Not reported | Not reported | Network staff identified a quality improvement coordinator within each intervention centre and maintained phone contact with that person during the remainder of the intervention period. | Face-to-face | Before & After | Not reported | Not reported |
| McClusky, 2016 | Printed educational materials, slides and other resources (eg list of transport available) | Given | Implications given | Not reported | Face-to-face + Educational materials | With | Experts | Clinicians |
| Mertens, 2015 | Case studies, role play and video endorsement from CEO. | Not reported | Implications given | Not reported | Face-to-face | With | Peers | Clinicians & Managers |
| Moher, 2001 | Guidelines | Not reported | Not reported | Not reported | Face-to-face | With | Experts | Clinicians |
| Mold, 2014 | Summaries of asthma guidelines and a toolkit containing an Asthma Control Test, the AsthmaAPGAR,33 and action plan templates in English and Spanish. | Supports local identification | Not reported | Not reported | Face-to-face + Educational materials | Not reported | Peers & QI Specialist | Clinicians |
| Mold, 2008 | Written report, a notebook with supporting materials | Not reported | Not reported | Not reported | Face-to-face + Educational materials | With | Experts & QI Specialist | Clinicians & Managers |
| Myers, 2004 | Reminder, slide presentation, one-page printed report, package of print materials and tailored letters to practices, | Given | Implications given | Not reported | Face-to-face + Educational materials | Not reported | Experts | Clinicians |
| Nilsson, 2001 | Educational material (available on request) | Not reported | Implications given | Not reported | Face-to-face + Educational materials | After | Experts | Clinicians |
| Palmer, 1985 | Relevant articles and survey | Supports local identification | Not reported | Not reported | Educational materials | After | Experts | Clinicians |
| Papadakis, 2018 | eHealth record tools and prompts; self-help materials for patients; patient follow-up support system. | Not reported | Not reported | Not reported | Face-to-face | With | Experts | Clinicians |
| Patel, 2018 | Not reported | Not reported | Implications given | Not reported | Face-to-face | With | Experts | Clinicians |
| Peiris, 2015 | Clinical decision support system | Supports local identification | Not reported | Not reported | Face-to-face | Not reported | Not reported | Clinicians |
| Pettersson, 2011 | Leaflet, handouts, written guideline | Not reported | Not reported | Not reported | Face-to-face | After | Experts & Peers | Clinicians |
| Price-Haywood, 2014 | Guidelines, website, narrative summaries of facilitator perceptions | Given | Not reported | Actors pretended to be patients, then revealed themselves as  actors and gave structured verbal feedback. | Face-to-face | After | QI Specialist | Clinicians |
| Quanbeck, 2018 | System flowchart and Plan-Do-Study-Act change forms, | Supports local identification | Not reported | Not reported | Face-to-face + Virtual (telephone + online) | Not reported | Experts | Clinicians |
| Quinley, 2004 | Chart stickers, patient education materials, medicare reimbursement rates and faxable order form | Given | Implications given | Not reported | Virtual (telephone) | Before & After | QI Specialist | Clinicians |
| Raasch, 2000 | None | Given | Implications given | Not reported | Virtual (telephone) + Educational materials | Not reported | Experts | Clinicians |
| Raja, 2015 | Physicians were given the medical record numbers of any patients for whom the CT examinations ordered were deemed nonadherent to evidence-based guidelines by CDS to facilitate individual chart review. | Given | Not reported | Not reported | Educational materials | Not reported | Experts | Clinicians |
| Rantz, 2001 | Quality Improvement manual and reference list of clinical practice standards | Supports local identification | Not reported | Not reported | Face-to-face + Virtual (telephone) + Educational materials | With | Experts | Clinicians & Managers |
| Rask, 2001 | Not reported | Not reported | Not reported | Not reported | Face-to-face | Not reported | QI Specialist | Clinicians |
| Ruangkanchanasetr , 1993 | Not reported | Given | Explored by data recipients | Not reported | Face-to-face | Not reported | Peers | Clinicians |
| Rubin, 2001 | Not reported | Given | Not reported | Not reported | Face-to-face | Not reported | Experts | Clinicians |
| Sauaia, 2000 | Not reported | Given | Not reported | Not reported | Face-to-face + Educational materials | After | Experts | Clinicians & Managers |
| Schectman, 2003 | Patient education resources, including a pamphlet and 10-minute educational video | Given | Implications given | Not reported | Face-to-face + Educational materials | Not reported | Experts | Clinicians |
| Schneider, 2007 | Not reported | Given | Explored by data recipients | Not reported | Not reported | Not reported | Peers | Clinicians |
| Scholes, 2006 | Newsletter and brightly coloured reminder labels | Given | Not reported | Not reported | Educational materials | Not reported | Peers | Not reported |
| Sinclair, 1982 | Not reported | Given | Not reported | Not reported | Face-to-face | With | Peers | Clinicians |
| Siriwardena, 2002 | Not reported | Given | Not reported | Not reported | Face-to-face | Not reported | Experts | Clinicians & Managers |
| Smith-Bindman, 2020 | Not reported | Given | Implications given | Not reported | Virtual (online) | After | Experts | Clinicians |
| Solomon, 2004 | Not reported | Given | Implications given | Not reported | Face-to-face | After | Experts | Clinicians |
| Sondergaard, 2005 | Price list of medications and patient handouts | Not reported | Implications given | n/a | Face-to-face + Educational materials | With | Experts | Clinicians |
| Soumerai, 1998 | Slide deck and educational brochures | Given | Implications given | n/a | Face-to-face + Educational materials | Not reported | Peers | Clinicians |
| Stewardson, 2016 | Written information about when hand hygiene needed | Not reported | Not reported | Individual level FF | Face-to-face | With | Experts | Clinicians |
| Tierney, 1986 | Reminders placed in notes | Not reported | Not reported | n/a | Virtual (online) | With | Virtual | Clinicians |
| Trietch, 2017 | Recommendations validated by clinical experts | Supports local identification | Not reported | N/A | Face-to-face | After | QI Specialist | Clinicians |
| Van De Velden, 2016 | Guidelines and patient educational booklets | Supports local identification | Implications given | n/a | Face-to-face + Educational materials | With | Not reported | Clinicians |
| van der Weijden, 1999 | Consultation registration forms, flow chart of guidelines and patient education leaflets | Given | Not reported | n/a | Face-to-face + Educational materials | After | Experts | Clinicians |
| Veninga, 1999 | Not reported | Given | Explored by data recipients | N/A | Face-to-face | Not reported | Peers & QI Specialist | Clinicians |
| Verstappen, 2003 | National guidelines | Not reported | Not reported | Discussed Bayesian decision rules to help them understand probability of false positives in low prevalence disorders. | Face-to-face + Educational materials | After | Peers | Clinicians |
| Vesrtappen, 2003 | Clinical guidelines | Given | Explored by data recipients | n/a | Face-to-face + Educational materials | After | Experts & Peers | Clinicians |
| Vingerhoets, 2001 | Manual described a number of ways to use the results including discussions with colleagues and assistants, detailed follow-up surveys among patients, and establishment of a patient panel. | Not reported | Not reported | n/a | Educational materials | With | Experts | Clinicians |
| Wahlstrom, 2003 | Not reported | Given | Implications given | N/A | Face-to-face | Not reported | QI Specialist | Clinicians |
| Walsh, 2007 | Not reported | Given | Explored by data recipients | n/a | Face-to-face + Virtual (online) | Not reported | Experts | Clinicians |
| Wang, 2018 | A clinical pathway document and guidelines integrated into care plan of eligible patients | Not reported | Not reported | n/a | Face-to-face + Educational materials | Before & With | Not reported | Clinicians |
| Wathne, 2018 | Presentation template which was modified by recipients | Given | Implications given | n/a | Face-to-face | Not reported | Experts & peers | Clinicians |
| Widden, 2018 | Not reported | Given | Implications given | n/a | Face-to-face | Before & After | Peers | Clinicians |
| Willis, 2020 | For each outreach visit, a practice-specific outreach pack was developed containing: audit report(s); a session outline; an action plan template that included space for noting current performance, setting a target, identifying who will do what and review date; and templates for assessing costs and benefits. | Not reported | Implications given | n/a | Face-to-face + Educational materials | After | QI Specialist | Clinicians |
| Wu, 2019 | Web-based support; patient educational materials and guidelines | Given | Implications given | n/a | Virtual (online) + Educational materials | Not reported | Experts | Not reported |

**Supplementary Table 1 continued:** A summary of the content and delivery of the included feedback facilitation interventions

| **Trial paper first author + year** | **Number of intervention arm sites** | **Number of people receiving the intervention at one time** | **Level of change sought** | **Tailoring** | **Was fidelity assessed** | **Reported fidelity adherence** | **Modification** | **Number of unreported TIDieR items** |
| --- | --- | --- | --- | --- | --- | --- | --- | --- |
| Althabe, 2019 | 13 | Not reported | Team | Not reported | Yes | 29-100 | Yes | 4 |
| Avery, 2010 | 36 | Not reported | Team | Not reported | No | Not applicable | Not reported | 7 |
| Awad, 2006 | 15 | 2 | Multi-team organisation | Not reported | No | Not applicable | Not reported | 5 |
| Ayieko, 2011 | 4 | Not reported | Team | Not reported | No | Not applicable | Not reported | 9 |
| Ayieko, 2019 | 2 | Not reported | Multi-team organisation | Not reported | Yes | Not reported | Not reported | 8 |
| Baker, 1997 | 8 | 8 | Team | No | No | Not applicable | No | 5 |
| Baldwin, 2010 | 16 | Not reported | Multi-team organisation | No | No | Not applicable | No | 5 |
| Barkun, 2013 | 21 | Not reported | Multi-team organisation | Yes | Yes | Not reported | Not reported | 5 |
| Bertoni, 2009 | 29 | Not reported | Team | No | Yes | 46 | Not reported | 6 |
| Bloos, 2017 | 19 | Not reported | Multi-team organisation | Not reported | No | Not applicable | Not reported | 8 |
| Bond, 2011 | 38 | Not reported | Team | Not reported | Yes | Not reported | Yes | 8 |
| Bonevski, 1999 | 19 | Not reported | Team | Yes | No | Not applicable | No | 3 |
| Borgiel, 1999 | 29 | 1 | Team | Not reported | No | Not applicable | Not reported | 6 |
| Bregnhoj, 2009 | 79 | Not reported | Team | Not reported | Not reported | Not reported | Not reported | 11 |
| Brown, 1994 | 24 | Not reported | Team | Yes | Yes | Not reported | Not reported | 7 |
| Chaillet, 2015 | 16 | Not reported | Multi-team organisation | Not reported | Not reported | Not reported | Not reported | 9 |
| Charrier, 2008 | 10 | Not reported | Team | Not reported | Not reported | Not reported | Not reported | 10 |
| Clarke, 2020 | 9 | Not reported | Other - Patients & multi-team organisation | Not reported | Yes | 80+ | Not reported | 6 |
| Cundill, 2015 | 24 | 5.5 | Team | No | Yes | 100 | Not reported | 3 |
| Curtis, 2011 | 6 | 3.5 | Team | No | Yes | 100 | Not reported | 4 |
| DeVore, 2015 | 73 | Not reported | Multi-team organisation | Yes | No | Not applicable | Not reported | 11 |
| Everett, 1983 | 13 | Not reported | Team | Not reported | No | Not applicable | Not reported | 7 |
| Fabbri, 2019 | 134 | Not reported | Wider system | No | No | Not applicable | Not reported | 6 |
| Filardo, 2009 | 23 | Not reported | Multi-team organisation | Not reported | Not reported | Not reported | Not reported | 9 |
| Foster, 2009 | 11 | 3 | Team | No | No | Not applicable | Not reported | 3 |
| Foy, 2004 | 13 | Not reported | Team | Not reported | No | Not applicable | Not reported | 7 |
| Frijling, 2002 | 62 | Not reported | Team | No | No | Not applicable | No | 5 |
| Frijling, 2003 | 62 | Not reported | Team | No | Yes | 96 | Not reported | 6 |
| Gilkey, 2014 | 61 | Not reported | Team | Not reported | Not reported | Not reported | Not reported | 7 |
| Gilkey, 2019 | 13 | 45 | team | Not reported | Yes | Not reported | Yes | 7 |
| Gjelstad, 2013 | 39 | Not reported | Team | Yes | Yes | Not reported | Not reported | 7 |
| Guadagnoli, 2000 | 18 | Not reported | Team | Yes | Not reported | Not reported | Not reported | 14 |
| Gude, 2016 | 18 | Not reported | Team | Not reported | Yes | 25-83.3 | Yes | 3 |
| Gulliford, 2019 | 41 | 1 | Team | No | No | Not applicable | No | 5 |
| Gullion, 1988 | 85 | 4 | Team | Not reported | No | Not applicable | No | 6 |
| Harris, 2015 | 16 | Not reported | Team | Not reported | No | Not applicable | Not reported | 7 |
| Hayes, 2001 | 14 | 1 | Multi-team organisation | Not reported | Yes | 100 | Not reported | 6 |
| Hayes, 2002 | 16 | 1 | Multi-team organisation | Not reported | Yes | 78 | Not reported | 8 |
| Hendryx, 1998 | 12 | Not reported | Multi-team organisation | Not reported | No | Not applicable | Not reported | 7 |
| Herbert, 2004 | 14 | 7 | Team | Not reported | No | Not applicable | Not reported | 8 |
| Hogg, 2008 | 27 | Not reported | Team | Yes | No | Not applicable | No | 2 |
| Houston, 2015 | 87 | 2 | Team | No | Yes |  | No | 3 |
| Huffman, 2018 | 63 | Not reported | Wider system | No | Yes | Not reported | Not reported | 7 |
| Huis, 2013 | 37 | Not reported | Team | No | No | Not applicable | No | 4 |
| Ivers, 2013 | 7 | 1 | Team | No | No | Not applicable | No | 3 |
| Kaufmann-Kolle, 2011 | 85 | 8 | Team | No | No | Not applicable | No | 7 |
| Kennedy, 2015 | 12 | 7 | Team | Yes | Yes | Not reported | Yes | 3 |
| Kiefe, 2001 | 48 | Not reported | Team | Not reported | Not reported | Not reported | Not reported | 9 |
| Kritchevsky, 2008 | 22 | 15 | Multi-team organisation | Yes | Yes | 100 | Not reported | 5 |
| Lakshminarayan, 2010 | 7 | Not reported | Multi-team organisation | No | No | Not applicable | Not reported | 5 |
| Lemelin, 2001 | 23 | Not reported | Team | No | Yes | 80 | No | 3 |
| Lesuis, 2018 | 20 | 20 | Multi-team organisation | No | Yes | 100 | No | 4 |
| Levi, 2020 | 10 | Not reported | Multi-team organisation | No | Yes |  | Not reported | 4 |
| Lopez-Picazo, 2011 | 69 | 1 | Team | Not reported | No | Not applicable | Not reported | 6 |
| Lynch, 2016 | 5 | Not reported | Team | No | Yes | 80 | Not reported | 3 |
| McCartney, 1997 | 14 | Not reported | Team | Not reported | No | Not applicable | Not reported | 10 |
| McClellan, 2004 | 21 | Not reported | Team | No | No | Not applicable | Not reported | 9 |
| McClusky, 2016 | 11 | Not reported | Team | Not reported | Yes | 82 | Not reported | 5 |
| Mertens, 2015 | 36 | Not reported | Team | No | Yes | 80-100 | No | 4 |
| Moher, 2001 | 21 | Not reported | Team | No | Yes | 95 | Yes | 7 |
| Mold, 2014 | 22 | Not reported | Team | No | Yes | 66 | Not reported | 6 |
| Mold, 2008 | 12 | Not reported | Team | Yes | Yes |  | No | 6 |
| Myers, 2004 | 120 | Not reported | Team | Yes | No | Not applicable | Not reported | 6 |
| Nilsson, 2001 | 40 | Not reported | Team | Not reported | No | Not applicable | Not reported | 8 |
| Palmer, 1985 | 16 | Not reported | Team | Not reported | No | Not applicable | Not reported | 8 |
| Papadakis, 2018 | 8 | Not reported | Team | Yes | No | Not applicable | Not reported | 5 |
| Patel, 2018 | Not reported | 9.8 | Team | No | Yes | 98 | Not reported | 6 |
| Peiris, 2015 | 30 | Not reported | Team | No | Yes | >50 | No | 9 |
| Pettersson, 2011 | 26 | 8 | Team | Not reported | No | Not applicable | Not reported | 4 |
| Price-Haywood, 2014 | Not reported | 1 | Team | Not reported | no | Not applicable | Not reported | 7 |
| Quanbeck, 2018 | 4 | 8.5 | Team | No | Yes | 69-92 | No | 3 |
| Quinley, 2004 | 811 | 1 | Team | Yes | No | Not applicable | No | 3 |
| Raasch, 2000 | 23 | Not reported | Team | No | No | Not applicable | No | 6 |
| Raja, 2015 | 1 | Not reported | Team | No | No | Not applicable | No | 7 |
| Rantz, 2001 | 36 | Not reported | Team | Yes | Yes |  | No | 6 |
| Rask, 2001 | 2 | Not reported | Team | Not reported | Not reported | Not applicable | No | 12 |
| Ruangkanchanasetr , 1993 | 1 | 9 | Team | Not reported | No | Not applicable | Not reported | 7 |
| Rubin, 2001 | 5 | Not reported | Multi-team organisation | Not reported | No | Not applicable | Not reported | 11 |
| Sauaia, 2000 | 9 | Not reported | Multi-team organisation | No | No | Not applicable | No | 6 |
| Schectman, 2003 | 14 | Not reported | Team | Not reported | Yes | 90 | Not reported | 8 |
| Schneider, 2007 | 6 | Not reported | Team | Not reported | No | Not applicable | Not reported | 11 |
| Scholes, 2006 | 12 | Not reported | Team | Not reported | No | Not applicable | Not reported | 10 |
| Sinclair, 1982 | 1 | 1 | Multi-team organisation | Not reported | No | Not applicable | Not reported | 6 |
| Siriwardena, 2002 | 15 | Not reported | Team | Not reported | No | Not applicable | Not reported | 8 |
| Smith-Bindman, 2020 | 129 | Not reported | Wider system | No | No | Not applicable | No | 4 |
| Solomon, 2004 | 1 | 21 | Team | Not reported | No | 95-100 | Yes | 5 |
| Sondergaard, 2005 | 14 | Not reported | Team | Not reported | Yes |  | Not reported | 8 |
| Soumerai, 1998 | 37 | Not reported | Multi-team organisation | Yes | No | Not applicable | No | 5 |
| Stewardson, 2016 | 46 | 3 | Other - Patients & multi-team organisation | No | Yes | 34 | No | 8 |
| Tierney, 1986 | 135 | Not reported | Team | No | No | Not applicable | No | 8 |
| Trietch, 2017 | 21 | 9 | Multi-team organisation | Yes | Not reported | Not reported | No | 4 |
| Van De Velden, 2016 | 45 | 3.5 | Team | No | Yes | 100 | No | 2 |
| van der Weijden, 1999 | 10 | Not reported | Team | No | No | Not applicable | No | 4 |
| Veninga, 1999 | 112 | Not reported | Team | Not reported | No | Not applicable | Yes | 6 |
| Verstappen, 2003 | 13 | 6 | Team | Not reported | No | Not applicable | Not reported | 5 |
| Vesrtappen, 2003 | 12 | 6.25 | Team | No | No | Not applicable | No | 2 |
| Vingerhoets, 2001 | 29 | Not reported | Team | No | No | Not applicable | No | 7 |
| Wahlstrom, 2003 | 12 | Not reported | Multi-team organisation | Not reported | Yes |  | Not reported | 8 |
| Walsh, 2007 | 7 | Not reported | Team | Yes | Yes |  | No | 6 |
| Wang, 2018 | 20 | 40 | Multi-team organisation | No | Yes | 40-100 | No | 4 |
| Wathne, 2018 | 3 | Not reported | Multi-team organisation | Yes | No | Not applicable | No | 6 |
| Widden, 2018 | 75 | Not reported | Wider system | Not reported | No | Not applicable | Not reported | 8 |
| Willis, 2020 | 67 | Not reported | Team | Yes | Yes | 47 | Not reported | 4 |
| Wu, 2019 | 101 | Not reported | Wider system | No | Yes | 38-100 | No | 8 |

**Supplementary materials 2:** The use of theory in included papers published since 2013

| **Name** | **Year** | **Illustrative Quote** | **Role of theory** |
| --- | --- | --- | --- |
| Althabe | 2019 | “Intervention strategies combined strategies shown to change providers’ behaviour with those based on the diffusion of innovation theory, with the provision of supplies for syphilis screening and treatment.” | Intervention design |
| Ayieko | 2019 | “To help create a bridge between this effort to identify the network intervention’s constituent parts and proposed mechanisms of action we have used the Behaviour Change Wheel framework … In essence, we are articulating an emerging theory of change from an insider perspective.”  “We wished to state our expectations of how the network may be operating in advance of this evaluation to allow for a more fruitful comparison.” | Intervention design; Evaluation |
| Clarke | 2020 | “Technology-assisted learning resources were also developed using motivational systems and instructional design theory” | Intervention Design |
| Cundill | 2015 | “A theoretical basis should increase internal coherence of an intervention and when specified explicitly in advance and should enable a better quality of evaluation. It should also enable wider applicability of results. We aimed to engage with theory to formulate the TACT intervention.”  “We set out an intensive one week agenda to (a) review findings from the formative research, empirical literature and behaviour change theory together to identify an approach to guide the intervention design; (b) identify potential strategies for achieving change by listing findings from the formative research, linking each to goals and selecting strategies from literature and theory for each; (c) refine this long list of potential intervention strategies using criteria agreed by the team; and (d) draft outlines of intervention activities and key content.”  “The theoretical approach to the leaflet was to go beyond didactic information-giving common to unsuccessful leaflets to a dialogue format in order to answer, through stories with characters that people could identify with, the questions being asked by people expected to take up rapid diagnostic tests”  “The workshops were designed to take place in small groups of three to eight health workers from neighbouring facilities, following the theory that communities of practice with trusted colleagues may support health worker behaviour change and that facilitated discussions based on observation and reflection on own practice with small groups of colleagues over time, such as Balint groups, can improve health worker decision-making and relationships with patients. The workshop activities and materials were designed to promote a learner-centred and interactive approach, and were based on adult learning steps of reflection on experience, conceptualisation and planning .” | Intervention Design |
| Fabbri | 2019 | “The study was a multigroup trial, whose design was motivated by a theory of change of how report cards might work—by leveraging providers’ pro-social motivation or by increasing citizens’ voice, community participation, and accountability—to improve coverage of maternal health services.” | Intervention Design |
| Gude | 2016 | During development of the QI plan content, additionally, the goal-setting theory, stating that feedback and well-specified goals are indeed a successful combination, was used. The theory emphasizes that people tend to be more committed to attaining a certain goal if they are involved in setting it, if goal attainment is seen as important, and if people believe they are capable of accomplishing it. To this end, each clinic needs to set up a local QI team with the responsibility to define, implement, and monitor a QI plan including concrete QI goals based on self-identified issues in the feedback reports.” | Intervention Design |
| Gulliford | 2019 | “McDermott et al identified theoretical components that relate directly to effective implementation in health-care settings, identifying aspects of social cognitive theory and self-determination theory as possible influences on GP prescribing behaviour.”  “Qualitative interviews in the previous study identified views that were consistent with self-determination theory.” | Intervention Design |
| Houston | 2015 | “Published in detail elsewhere, a facilitation-based program, guided by the Promoting Action on Research Implementation in Health Services (PARIHS) framework, was instituted to support implementation referrals in both the paper and practice portal arms. PARIHS provides a conceptual framework that incorporates various  influences that interact within implementation. When used as a guiding framework, PARIHS can highlight important evidence and contextual factors that may influence an implementation effort and can inform the development of a suitable facilitation-based implementation strategy.” | Intervention Design |
| Huis | 2013 | “The team and leaders-directed strategy was also aimed at addressing barriers at team level by focussing on social influence in groups and strengthening leadership. The unique contribution of this strategy was built upon the social learning theory (Bandura, 1986), social influence theory (Mittman et al., 1992), theory on team effectiveness (Shortell et al., 2004; West, 1990) and leadership theory (Øvretveit, 2004). The team and leaders-directed strategy included all elements of the state-of-the-art strategy (a–d) supplemented with (e) gaining active commitment and initiative of ward management (f) modelling by informal leaders at the ward, and (g) setting norms and targets within the team.” | Intervention Design |
| Ivers | 2013 | “According to Goal-Setting Theory, those who are dissatisfied with their performance will develop a change in behaviour if they are committed to the goal and if they meet a threshold level of self-efficacy for that task. Bandura explains that people are more likely to try to accomplish a goal if they believe their efforts will be successful.”  “In the context of feedback and goals, these plans may increase goal-directed behaviours by increasing self-efficacy. Action plans can also facilitate success by increasing goal-commitment to overcome barriers such  as distraction or fatigue; implementation plans in particular seem to increase goal-directed behaviours. Implementation intentions are developed through if/then statements, wherein the participant must connect a situation (if) with a behavioural response (then). With some effort (i.e., considering and writing down the plan), the connection made between contextual cues and an action plan can become automatic, thereby increasing goal attainment without conscious intent…. The assumption is that healthcare providers intend to provide consistently high quality of care, but are uncertain how to change their behaviours to accomplish this feat. Therefore, an intervention aiming to close this intention-behaviour-gap could be very effective.” | Intervention Design |
| Kennedy | 2015 | “The design and implementation of our 12-month, multifaceted intervention was founded on the Canadian Institutes of Health Research Knowledge-to-Action cycle [21], described in the study protocol [19].” | Intervention design |
| Levi | 2020 | “Elements were developed in accordance with the Behaviour Change Wheel method and strategies regarding; staff capability, opportunity and motivation to perform the desired behavior; and behaviour change techniques such as education, training, environmental restructuring, modelling, and enablement.” | Intervention Design |
| Lynch | 2016 | The multifaceted intervention was developed using the implementation of change theoretical model” | Intervention Design |
| Mold | 2014 | “Assumptions behind PF [practice facilitation] are that many practices are inadequately resourced, lack the experience and skills required, and are so unique that each must implement innovations differently. Relationships between practice facilitators and practice staff appear to be critical to success, resembling the Cooperative Extension, where agents develop relationships with families to facilitate implementation of evidence-based farming practices” | Intervention Design |
| Papadakis | 2018 | “Designed to influence the following 4 factors known to affect self‑efficacy: (1) skills targeted clinician self-efficacy through training, (2) personal experience, (3) modelling of behaviors, and (4) positive social or environmental supports” | Intervention Design |
| Van De Velden | 2016 | “GP education was not specifically based on behavioural change frameworks but contained elements we considered useful and informative for GPs.” | Intervention Design |
| Willis | 2020 | “Targeting implementation strategies according to determinants of adherence (also known as barriers, enablers, or facilitators that influence or affect behaviour) may improve their effectiveness. The Theoretical Domains Framework (TDF) offers a structured approach for exploring the perceptions of those whom are targets for the intervention, whilst the Behaviour Change Taxonomy outlines 16 categories of 93 specific, theoretically informed or evidence-based behaviour change techniques (BCTs) that are hypothesised to change behaviour”  Stages:  Stage 1: selecting delivery mechanisms.  Stage 2: identifying candidate BCTs.  “Team members with experience of applying behavioural theories to implementation strategies independently mapped the 12 determinants from the TDF to one or more of the 16 broad BCT categories and then to individual BCTs using an electronic spreadsheet. Results were collated and any BCT category nominated by three or more researchers was considered eligible. The team discussed discrepancies until consensus was agreed. We aimed to generate an inclusive list of ‘candidate’ change techniques. A matrix was produced to indicate BCTs with the potential to target one or more Theoretical determinants.”  Stage 3: identifying and prioritising relevant theoretical determinants of behaviour.  Stage 4: designing intervention content.  Stage 5: piloting intervention content and refinement.  Stage 6: verification of BCTs included within implementation packages. | Intervention Design |

**Supplementary materials 3:** A Sankey chart to describe the identification of influences (co-produced, sought by feedback recipients or given by study team) and the identification of improvement strategies within included studies.

| **Identification of influences** | 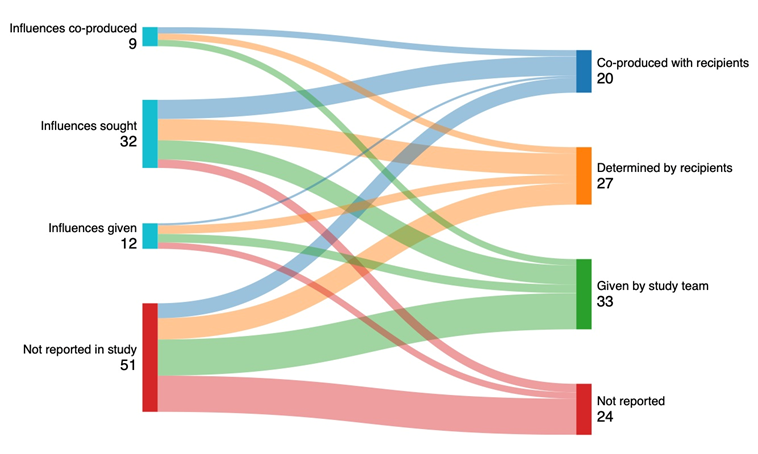 | **Identification of strategies** |
| --- | --- | --- |

**Supplementary materials 4:** A Venn diagram to illustrate who delivered feedback facilitation

Virtual (N=2)

Experts & QI specialists (n=3)

Experts & Peers (n=8)

Peers & QI specialists (n=4)

**Supplementary materials 5:** A graph to compare the numbers of recipients per site with number of intervention arm sites


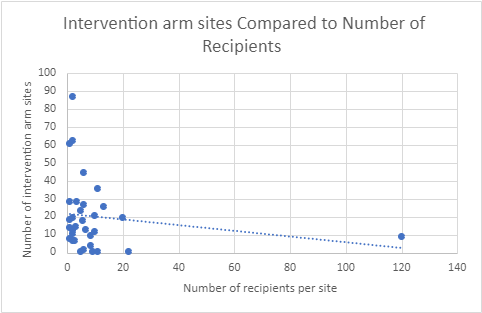


**Supplementary materials 6:** A box plot of the number of people receiving the intervention at one time by setting


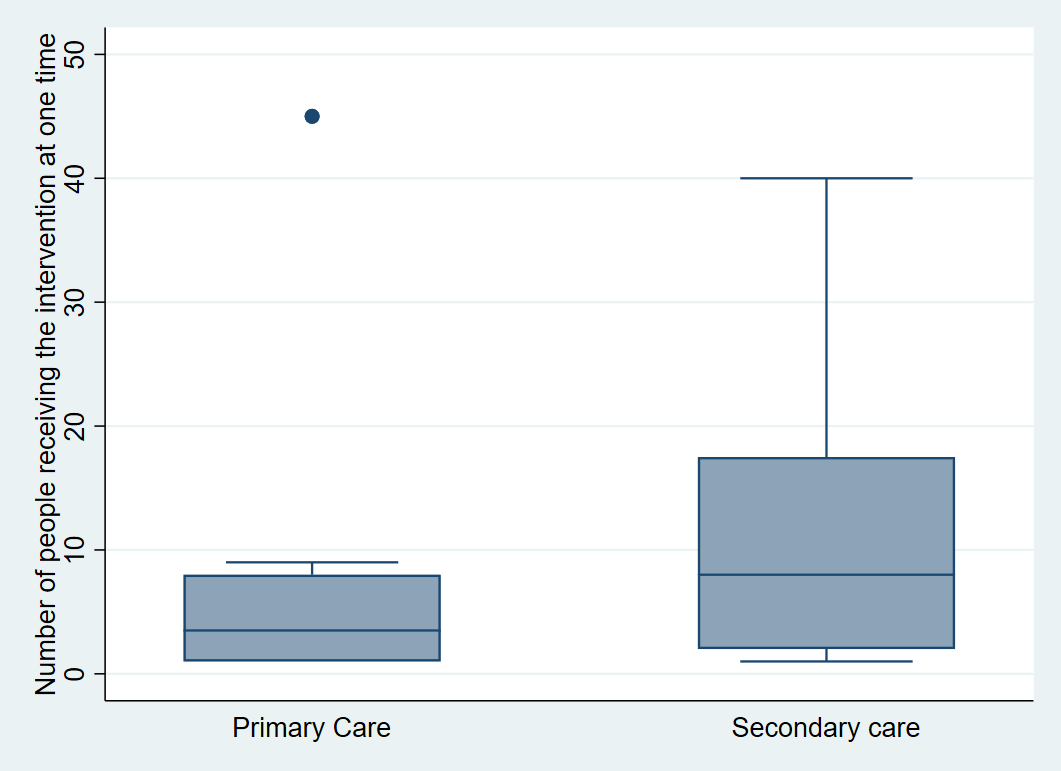


**Supplementary materials 7:** Exploring differences between primary and secondary care

|  | Primary care | Secondary care |
| --- | --- | --- |
| **Number of strategies** | | |
| 1 | 8 | 3 |
| 2 | 14 | 12 |
| 3 | 10 | 11 |
| 4 | 14 | 6 |
| 5 | 6 | 6 |
| 6 | 1 | 3 |
| 7 | 0 | 1 |
| 8 | 1 | 0 |
| **Exploration of influences** | | |
| Influences sought by recipients | 18 | 12 |
| Influences given | 4 | 6 |
| Co-produced | 4 | 5 |
| **Identification of actions** | | |
| Actions determined by recipients | 12 | 12 |
| Actions given | 16 | 15 |
| Co-produced | 12 | 8 |
| **Identification of implications** | | |
| Explored implications | 4 | 3 |
| Implications given | 19 | 15 |
| **Who delivered** | | |
| Delivered by experts | 20 | 18 |
| Peers | 16 | 8 |
| QI specialist | 8 | 5 |

**Supplementary materials 8:** ERIC Implementation strategies not identified within feedback facilitation interventions

1. Alter incentive/allowance structures;
2. Alter patient/consumer fees;
3. Assess for readiness and identify barriers and facilitators;
4. Audit and provide feedback;
5. Build a coalition;
6. Capture and share local knowledge;
7. Centralize technical assistance;
8. Change accreditation or membership requirements;
9. Change liability laws;
10. Change service sites;
11. Conduct local needs assessment;
12. Conduct ongoing training;
13. Create new clinical teams;
14. Create or change credentialing and/or licensure standards;
15. Develop academic partnerships;
16. Develop an implementation glossary;
17. Develop disincentives;
18. Develop resource sharing agreements;
19. Distribute educational materials;
20. Facilitate relay of clinical data to providers;
21. Fund and contract for the clinical innovation;
22. Identify early adopters;
23. Increase demand;
24. Intervene with patients/consumers to enhance uptake and adherence;
25. Involve patients/consumers and family members;
26. Make billing easier;
27. Make training dynamic;
28. Mandate change;
29. Obtain and use patients/consumers and family feedback;
30. Organize clinician implementation team meetings;
31. Place innovation on fee for service lists/formularies;
32. Promote adaptability;
33. Provide ongoing consultation;
34. Revise professional roles;
35. Shadow other experts;
36. Stage implementation scale up;
37. Start a dissemination organization;
38. Use advisory boards and workgroups;
39. Use an implementation advisor;
40. Use capitated payments;
41. Use data experts;
42. Use data warehousing techniques;
43. Use mass media;
44. Use other payment schemes;
45. Use train-the-trainer strategies;
46. Visit other sites;
47. Work with educational institutions;
