# Supplementary material for: Exploring the content and delivery of feedback facilitation co-interventions: a systematic review
Source: Implement Sci. 2024 May 28;19:37. doi: 10.1186/s13012-024-01365-9 (PMC11134935; doi:10.1186/s13012-024-01365-9)
Supplement: Supplementary file 2 — Supplementary Material 2. [file 13012_2024_1365_MOESM2_ESM.docx]

**Appendix A: Extraction guidance notes**

1. **Author**

The surname of the first author

1. **Year**

The year published

1. **Companion papers**

Citation

1. **Brief name;**

Record the feedback facilitation intervention name as it is given in the paper, or if not stated, summarised by reviewer in the next column.

1. **Brief description by extractors**
2. **Rationale or theory;**

The given name of the theory; or Description of rationale given; or None

1. **What materials in the co-intervention**

Describe materials (e.g. a framework to analyse determinants, PowerPoint presentation describing QI methods, action plan)

The next 9 items (J to R) relate to the target behaviours, that is, what does the intervention seek to implement in the recipients of feedback facilitation:

1. **Identification of priorities from data (Given / Supports local identification / No / Not applicable/Not recorded)**

Where the audit measures performance on a number of items of care, the feedback facilitation might direct towards one item, might ask recipients to select a priority, might not seek a priority. Alternatively, there might only be one data item.

1. **Describe prioritisation procedure**

Use language as close to that in the text as possible whilst making sense as stand alone entry

1. **Does the intervention explicitly seek to address inequalities? (Y/N)**

Are any of the prioritised interventions explicitly focused on reducing health inequalities seen in the population receiving the intervention? Those health inequalities may by based on socio-economic factors, geography, specific protected characteristics, or socially excluded groups and seek to address, for example, differences in health outcomes, access to healthcare, quality of care, engagement in risky behaviour, or wider determinants of health.

1. **Explore influences upon performance (Influences or causes given, sought by data recipients or Not recorded)**

Does the co-intervention seek to implement work to explore influences upon performance, including barriers/facilitators to implementation? (e.g. Used Theoretical domains framework [182] to identify determinants). ‘Influences sought by data recipient’s includes where a potential list of influences is given by the intervention deliverers, from which recipients are to select based upon local knowledge/analysis.

1. **Describe procedure to explore influences**

Use language as close to that in the text as possible whilst making sense as stand alone entry

1. **How are implementation strategies determined**

Describe the process by which implementation strategies are determined (for example, they are given by the study team, they are selected by data recipients) or n/a

1. **Information on implications of performance given, explored by data recipients or n/a**

Does the co-intervention explore reasons for changing current performance, either by giving implications or supporting participants to consider implications from current performance? (e.g. Suggest the participants describe impact of performance upon patient outcomes that are not already part of the feedback, length of stay, cost, reputation)

1. **Describe procedure for exploring implications**

Use language as close to that in the text as possible whilst making sense as stand alone entry

1. **Other**

Describe other target behaviours that the feedback facilitation seeks to implement. Please use language from the article in a way that makes sense as stand alone entry. For example, some papers describe implementation of plan-do-study-act (PDSA). Please use this language and record under ‘OTHER’, the synthesis can consider extent to which overlap with, for example, exploring influences and select strategies is part of the plan.

The next 9 items (S to Y) relate to the delivery of feedback facilitation, that is, how do the intervention deliverers seek to implement the target behaviours:

1. **How delivered**

Is the intervention delivered Face-to-face training +/- Virtual training +/- Training +/-educational materials +/-Facilitation +/-other

1. **Frequency**

How often did those delivering the co-intervention have contact with the recipients

1. **Duration**

How long (in days, hours and minutes) was the contact with the recipients?

1. **Timing**

Number of weeks/months before or after feedback (expressed as, for example, - 4weeks, if before the audit feedback is given)

1. **Who delivered the co-intervention**

This means within the study, not the intervention recipients delivering to others. Use language as close to that in the text as possible whilst making sense as stand alone entry

1. **Who were the co-intervention recipients**

Use language as close to that in the text as possible whilst making sense as stand alone entry

1. **Number of recipients per site**

How many people received the feedback facilitation per site? (e.g. training to three hospital clinicians forming the ‘core team’ responding to an audit (n=3); outreach visit to a general practice team meeting with eight people present (n=8))

1. **Number of intervention arm sites**

In the intervention arm only

1. **Number of people receiving the intervention at one time**

Describe how many received the intervention together (e.g. a workshop of 40 people from 20 sites)

1. **Setting (e.g. hospital)**

Describe the type of site, rather than where the intervention was delivered (e.g. hospital not lecture theatre)

**AA. Organizational Level of change sought**

- Level 1 = Team E.g. General practice, intensive care wards
- Level 2 = Multi-team organisation e.g. Hospital or group of practices
- Level 3 = Wider system e.g. geographical region or state
- Other
- Not recorded

Note: We are excluding feedback facilitation where the intervention exclusively provides clinical supervision about individual patients

**AB. Tailoring**

Were there planned local adaptation to recipients (e.g. three sites received intervention virtually to reduce travel time), if so, how. Unplanned tailoring should be captured in fidelity columns.

**AC. Is there a description of how fidelity in delivery was assessed (Y/N/Not recorded)**

Was the fidelity of how the co-intervention was delivered assessed.

**AD. Fidelity in delivery adherence (Describe)**

Fidelity of delivery is defined as, “extent to which intervention content is delivered

as intended during the intervention period” (Lorencatto et al, 2016; p4). Where fidelity was assessed, did the assessment find that it was delivered with fidelity? For example, facilitation was planned to be face-to-face but amended it at four sites to be virtual. Use language as close to that in the text as possible whilst making sense as stand alone entry

**AE. Modification of the intended procedures by recipient (Describe)**

Did recipients modify the procedures/target behaviours from those delivered in the co-intervention. For example, that feedback facilitation trained participants to review their data and identify local priorities, but the participants identified priorities based upon national priorities. Alternatively, the feedback facilitation guided people to use plan-do-study-act but the participants didn’t measure (i.e. study) whether the changes they made had an effect.

**AF. Extent to which the intervention is solely feedback facilitation ? (FF Alone/Mixed/ Unclear)**

Does the intervention purely seek to increase the capability, motivation or opportunity to respond to the data, or are there additional components (e.g. to provide educational content about the clinical domain) or is it unclear. Note: Feedback Facilitation Alone includes the delivery of audit and feedback.

Post-review note: Additional definitions are provided in the Text Box 1 and in the TIDieR paper [10] upon which the extraction tool is based.

**Appendix B: PRISMA checklist**

i) for abstracts

| **Section and Topic** | **Item #** | **Checklist item** | **Reported (Yes/No)** |
| --- | --- | --- | --- |
| **TITLE** | | |  |
| Title | 1 | Identify the report as a systematic review. | Y |
| **BACKGROUND** | | |  |
| Objectives | 2 | Provide an explicit statement of the main objective(s) or question(s) the review addresses. | Y |
| **METHODS** | | |  |
| Eligibility criteria | 3 | Specify the inclusion and exclusion criteria for the review. | Y |
| Information sources | 4 | Specify the information sources (e.g. databases, registers) used to identify studies and the date when each was last searched. | Y |
| Risk of bias | 5 | Specify the methods used to assess risk of bias in the included studies. | N/A |
| Synthesis of results | 6 | Specify the methods used to present and synthesise results. | Y |
| **RESULTS** | | |  |
| Included studies | 7 | Give the total number of included studies and participants and summarise relevant characteristics of studies. | Y |
| Synthesis of results | 8 | Present results for main outcomes, preferably indicating the number of included studies and participants for each. If meta-analysis was done, report the summary estimate and confidence/credible interval. If comparing groups, indicate the direction of the effect (i.e. which group is favoured). | Y |
| **DISCUSSION** | | |  |
| Limitations of evidence | 9 | Provide a brief summary of the limitations of the evidence included in the review (e.g. study risk of bias, inconsistency and imprecision). | N |
| Interpretation | 10 | Provide a general interpretation of the results and important implications. | Y |
| **OTHER** | | |  |
| Funding | 11 | Specify the primary source of funding for the review. | N |
| Registration | 12 | Provide the register name and registration number. | Y |

ii) For systematic reviews

| **TITLE** | | |  |
| --- | --- | --- | --- |
| Title | 1 | Identify the report as a systematic review. | Title |
| **ABSTRACT** | | |  |
| Abstract | 2 | See the PRISMA 2020 for Abstracts checklist. | Supp Materials |
| **INTRODUCTION** | | |  |
| Rationale | 3 | Describe the rationale for the review in the context of existing knowledge. | 3 |
| Objectives | 4 | Provide an explicit statement of the objective(s) or question(s) the review addresses. | 3 |
| **METHODS** | | |  |
| Eligibility criteria | 5 | Specify the inclusion and exclusion criteria for the review and how studies were grouped for the syntheses. | 4 |
| Information sources | 6 | Specify all databases, registers, websites, organisations, reference lists and other sources searched or consulted to identify studies. Specify the date when each source was last searched or consulted. | 5 |
| Search strategy | 7 | Present the full search strategies for all databases, registers and websites, including any filters and limits used. | 4 |
| Selection process | 8 | Specify the methods used to decide whether a study met the inclusion criteria of the review, including how many reviewers screened each record and each report retrieved, whether they worked independently, and if applicable, details of automation tools used in the process. | 4 |
| Data collection process | 9 | Specify the methods used to collect data from reports, including how many reviewers collected data from each report, whether they worked independently, any processes for obtaining or confirming data from study investigators, and if applicable, details of automation tools used in the process. | 4 |
| Data items | 10a | List and define all outcomes for which data were sought. Specify whether all results that were compatible with each outcome domain in each study were sought (e.g. for all measures, time points, analyses), and if not, the methods used to decide which results to collect. | Appendix A |
|  | 10b | List and define all other variables for which data were sought (e.g. participant and intervention characteristics, funding sources). Describe any assumptions made about any missing or unclear information. | Appendix A |
| Study risk of bias assessment | 11 | Specify the methods used to assess risk of bias in the included studies, including details of the tool(s) used, how many reviewers assessed each study and whether they worked independently, and if applicable, details of automation tools used in the process. | 4 |
| Effect measures | 12 | Specify for each outcome the effect measure(s) (e.g. risk ratio, mean difference) used in the synthesis or presentation of results. | N/A |
| Synthesis methods | 13a | Describe the processes used to decide which studies were eligible for each synthesis (e.g. tabulating the study intervention characteristics and comparing against the planned groups for each synthesis (item #5)). | 4 |
|  | 13b | Describe any methods required to prepare the data for presentation or synthesis, such as handling of missing summary statistics, or data conversions. | 4 |
|  | 13c | Describe any methods used to tabulate or visually display results of individual studies and syntheses. | 4 |
|  | 13d | Describe any methods used to synthesize results and provide a rationale for the choice(s). If meta-analysis was performed, describe the model(s), method(s) to identify the presence and extent of statistical heterogeneity, and software package(s) used. | 4 |
|  | 13e | Describe any methods used to explore possible causes of heterogeneity among study results (e.g. subgroup analysis, meta-regression). | 4 |
|  | 13f | Describe any sensitivity analyses conducted to assess robustness of the synthesized results. | N/A |
| Reporting bias assessment | 14 | Describe any methods used to assess risk of bias due to missing results in a synthesis (arising from reporting biases). | N/A |
| Certainty assessment | 15 | Describe any methods used to assess certainty (or confidence) in the body of evidence for an outcome. | N/A |

| **RESULTS** | | |  |
| --- | --- | --- | --- |
| Study selection | 16a | Describe the results of the search and selection process, from the number of records identified in the search to the number of studies included in the review, ideally using a flow diagram. | 5 |
|  | 16b | Cite studies that might appear to meet the inclusion criteria, but which were excluded, and explain why they were excluded. | N/A |
| Study characteristics | 17 | Cite each included study and present its characteristics. | Table 1 |
| Risk of bias in studies | 18 | Present assessments of risk of bias for each included study. | Reference Cochrane review |
| Results of individual studies | 19 | For all outcomes, present, for each study: (a) summary statistics for each group (where appropriate) and (b) an effect estimate and its precision (e.g. confidence/credible interval), ideally using structured tables or plots. | Reference Cochrane review |
| Results of syntheses | 20a | For each synthesis, briefly summarise the characteristics and risk of bias among contributing studies. | N/A |
|  | 20b | Present results of all statistical syntheses conducted. If meta-analysis was done, present for each the summary estimate and its precision (e.g. confidence/credible interval) and measures of statistical heterogeneity. If comparing groups, describe the direction of the effect. | Results section |
|  | 20c | Present results of all investigations of possible causes of heterogeneity among study results. | Results section |
|  | 20d | Present results of all sensitivity analyses conducted to assess the robustness of the synthesized results. | N/A |
| Reporting biases | 21 | Present assessments of risk of bias due to missing results (arising from reporting biases) for each synthesis assessed. | N/A |
| Certainty of evidence | 22 | Present assessments of certainty (or confidence) in the body of evidence for each outcome assessed. | Results section |
| **DISCUSSION** | | |  |
| Discussion | 23a | Provide a general interpretation of the results in the context of other evidence. | 15 |
|  | 23b | Discuss any limitations of the evidence included in the review. | 16 |
|  | 23c | Discuss any limitations of the review processes used. | 17 |
|  | 23d | Discuss implications of the results for practice, policy, and future research. | 16 |
| **OTHER INFORMATION** | | |  |
| Registration and protocol | 24a | Provide registration information for the review, including register name and registration number, or state that the review was not registered. | 2 |
|  | 24b | Indicate where the review protocol can be accessed, or state that a protocol was not prepared. | 2 |
|  | 24c | Describe and explain any amendments to information provided at registration or in the protocol. | 16 |
| Support | 25 | Describe sources of financial or non-financial support for the review, and the role of the funders or sponsors in the review. | 19 |
| Competing interests | 26 | Declare any competing interests of review authors. | 19 |
| Availability of data, code and other materials | 27 | Report which of the following are publicly available and where they can be found: template data collection forms; data extracted from included studies; data used for all analyses; analytic code; any other materials used in the review. | 19 |
